# LMNB1 reduction is a potential therapeutic strategy in a mouse model of autosomal dominant leukodystrophy

**DOI:** 10.1172/jci.insight.207889

**Published:** 2026-08-11

**Authors:** Nathan Herdman, Kaveh Moradi, Bruce Nmezi, Anushe Munir, Krizchelle A. Magtoto, Fang Liu, Mara Sullivan, Xuemei Zeng, Thomas K. Karikari, Quasar S. Padiath

**Affiliations:** 1 Department of Human Genetics, School of Public Health,; 2 Center for Biologic Imaging, and; 3 Department of Psychiatry, School of Medicine, University of Pittsburgh, Pittsburgh, Pennsylvania, USA.; 4 Biofluid Biomarker Laboratory, Western Psychiatric Hospital, University of Pittsburgh Medical Center, Pittsburgh, Pennsylvania, USA.; 5 Alzheimer’s Disease Research Center and; 6 Department of Neurobiology, School of Medicine, University of Pittsburgh, Pittsburgh, Pennsylvania, USA.

**Keywords:** Genetics, Neuroscience, Demyelinating disorders, Mouse models, Neurodegeneration

## Abstract

Autosomal dominant leukodystrophy (ADLD) is a fatal adult-onset CNS demyelinating disorder for which no treatment exists. The majority of ADLD cases are caused by duplications of the lamin B1 *(LMNB1*) gene, resulting in increased LMNB1 expression. While reducing LMNB1 levels represents a logical therapeutic strategy, its efficacy has not been previously demonstrated in any in vivo model. Mouse models with oligodendrocyte-specific human LMNB1 (hLMNB1) overexpression recapitulate salient features of ADLD. Using a modified version of this model, where hLMNB1 can be inducibly downregulated, we demonstrated that hLMNB1 reduction can prevent or substantially ameliorate disease progression. Therapeutic effects were maximized when hLMNB1 reduction was induced before expected symptom onset, resulting in improvements in behavioral, biochemical, histopathological, and survival measures relative to those of untreated animals. Reducing hLMNB1 levels after symptom onset led to improved survival but mixed results for other disease phenotypes. In addition, we identified potential biomarkers that track disease progression. Furthermore, we demonstrated that near-complete knockdown of murine LMNB1 expression in adulthood did not result in any overt CNS phenotype. Together, these results provide a proof of concept supporting LMNB1 reduction as a therapeutic strategy and offer a rationale for treatments aimed at lowering levels of this protein in ADLD.

## Abstract 2

Down regulation of the LMNB1 protein can improve disease outcomes in a mouse model of the fatal CNS demyelinating disorder, Autosomal Dominant Leukodystrophy.

## Introduction

Autosomal dominant leukodystrophy (ADLD; OMIM #169500) is an ultra-rare adult-onset neurological disease characterized by progressive CNS demyelination and age-dependent motor decline (1). Clinical symptoms typically present in the fourth to fifth decade of life, beginning with autonomic dysfunction and progressing to total limb paralysis (2, 3). After symptom onset, affected individuals normally succumb to the disease within 10–20 years (2). Currently, no treatment or cure exists for ADLD, with symptom management being the only option (1, 4).

ADLD is caused by genomic mutations involving the lamin B1 *(LMNB1*) gene, with the most common ADLD-causing mutation being a tandem duplication encompassing *LMNB1* (3, 5–7). In addition, deletions upstream of *LMNB1* are associated with a variant form of ADLD (3, 7–10). In both cases, disease pathogenesis is thought to be driven by LMNB1 overexpression. We recently demonstrated that these disease-causing structural variants disrupt the interaction between the *LMNB1* gene and an upstream, oligodendrocyte-specific silencer element, resulting in oligodendrocyte-specific LMNB1 overexpression and providing a potential mechanism for the cell-type specificity of the demyelination phenotype (10). However, it remains unclear whether ADLD is primarily driven by early LMNB1 overexpression or by continued expression throughout life.

LMNB1 is a key structural component of the nuclear lamina, a network of intermediate filament proteins located along the inner nuclear membrane (11, 12). In addition to providing structural support to the nucleus, the nuclear lamina participates in multiple cellular processes, including chromatin and genome organization through lamina-associated domains, DNA repair, and transcriptional regulation (12). LMNB1 is widely expressed at varying levels across cell types and tissues, with expression decreasing during cellular differentiation and with age in tissues such as the hippocampus and skin (13–15). During development, LMNB1 expression is high and plays a crucial role in organogenesis and the migration of neurons, as demonstrated by the perinatal lethality of forebrain-specific *Lmnb1* knockout mice, which exhibit abnormal cortical neuron layering and reduced forebrain size (16–18).

We have previously generated a transgenic (TG) mouse model in which a FLAG-tagged human LMNB1 (hLMNB1) construct was overexpressed specifically in oligodendrocytes using a proteolipid protein 1 (*Plp1*) promoter, a gene highly expressed in mature oligodendrocytes (19, 20). The mouse and human LMNB1 proteins have a 97% sequence identity (human LMNB1, UniProt P20700; mouse LMNB1, UniProt P14733), which is considerably higher than the average conservation between mouse and human proteins (~85%) (21). Given this high degree of conservation, the two proteins are expected to be functionally equivalent. These TG mice recapitulated several key features of ADLD, including age-dependent motor decline, limb paralysis, CNS demyelination, vacuolar degeneration, and reduced survival (19). In contrast, mice with LMNB1 overexpression targeted to astrocytes or neurons did not exhibit any disease phenotypes (20). Nevertheless, astrocytes and neurons may still contribute to ADLD pathogenesis, as prior in vitro studies have reported increased cellular abnormalities following LMNB1 overexpression in astrocytes (22). Complementing these in vivo findings, cell culture work with mouse embryonic fibroblasts overexpressing LMNB1, as well as fibroblasts from patients with ADLD, demonstrated increased nuclear abnormalities, which were reduced upon downregulation of LMNB1 expression (23–26). However, the relationship between these nuclear abnormalities and the disease phenotype is unclear. Because increased LMNB1 expression underlies disease pathology, therapies that reduce LMNB1 levels would be a logical and attractive therapeutic strategy. However, to date, there have been no demonstrations in any *in vivo* preclinical model that reducing LMNB1 levels has a therapeutic effect, even as a proof of concept.

To address this gap, we generated a mouse model in which hLMNB1 overexpression could be inducibly ablated and investigated whether reducing hLMNB1 would be an effective therapeutic strategy. This also allowed us to determine if the timing of hLMNB1 reduction influenced disease outcomes. Our results indicate that reducing hLMNB1 in adult ADLD TG mice halted or slowed ADLD pathology. TG mice with reduced hLMNB1 expression before the onset of expected symptoms showed the greatest improvement and, in most cases, were indistinguishable from WT mice. When overexpression was inhibited after the onset of symptoms, disease mitigation was limited but overall survival was improved. Additionally, we demonstrate that a ubiquitous knockout of murine LMNB1 in adult mice resulted in no discernible phenotypes, providing a therapeutic window of safety for reducing LMNB1 levels. Together, these findings provide a proof of concept that targeting LMNB1 overexpression represents a promising therapeutic strategy for ADLD-affected individuals and highlight the importance of early intervention.

## Results

### Plp-FLAG-LMNB1-flox mice recapitulate ADLD pathology and are capable of inducible hLMNB1 reduction.

*Plp-FLAG-LMNB1-*flox TG mice were generated carrying loxP sites flanking the hLMNB1 construct driven by the mouse *Plp1* promoter (TG-flox) (Supplemental Figure 1A; supplemental material available online with this article; https://doi.org/10.1172/jci.insight.207889DS1). Prior work in the TG mouse model, which used the same cassette but lacked the loxP sites, demonstrated specific overexpression of hLMNB1 in mature oligodendrocytes and recapitulation of the ADLD phenotype (19).

**Figure 1:**
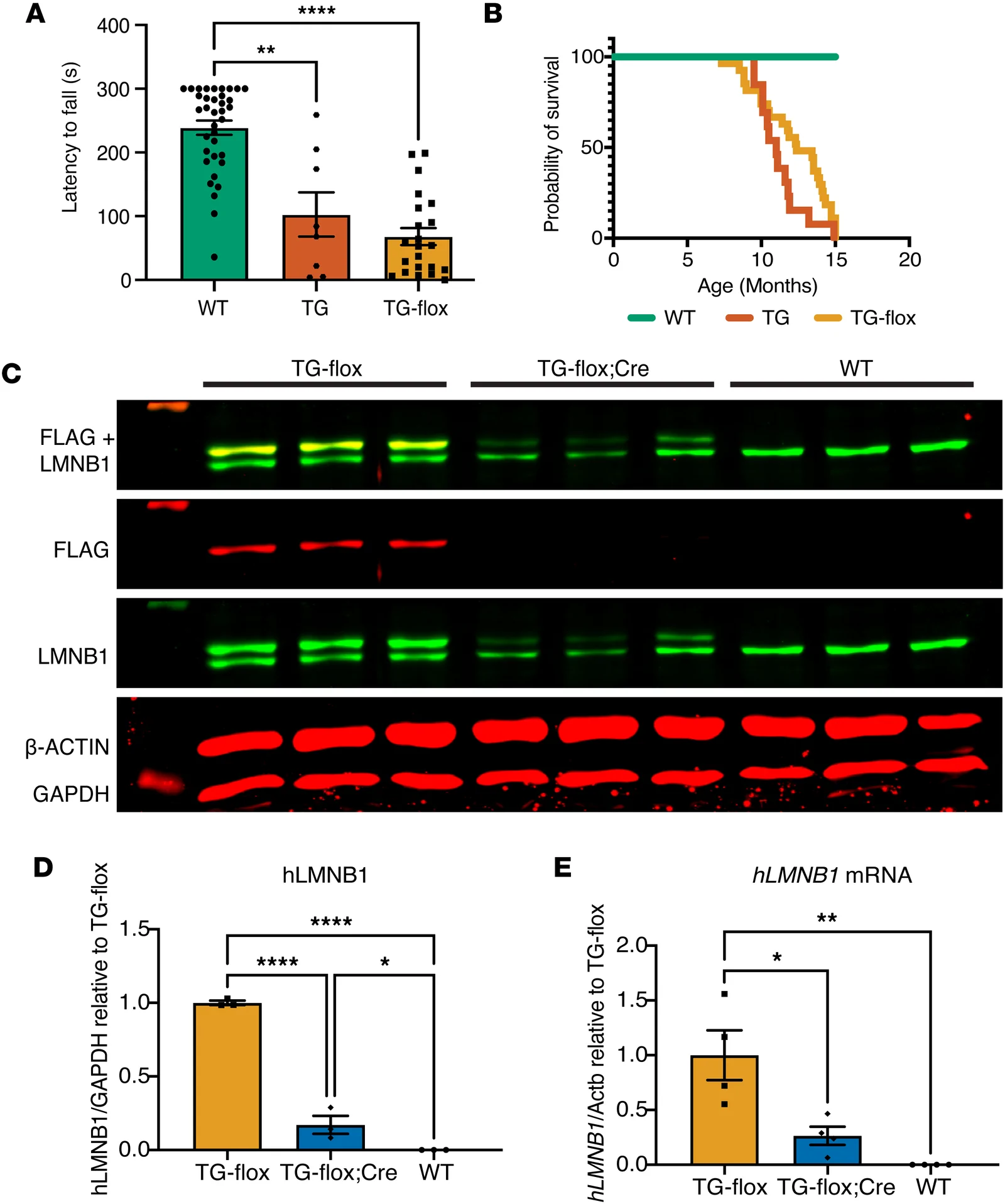
ADLD mouse models are comparable, and tamoxifen-induced reduction of hLMNB1 is possible when crossed with a *Plp1*-cre/ER^T^ line. (**A**) Rotarod of ADLD mouse lines at 7 months of age. *n* ≥ 8 per group. (**B**) Kaplan-Meier survival curves for ADLD mouse model lines. Survival curves differed significantly by log-rank (Mantel-Cox) test (*P* < 0.0001). Pairwise log-rank tests with correction for multiple comparisons showed reduced survival in WT vs. TG (adjusted *P* = 0.0003) and WT vs. TG-flox (adjusted *P* = 0.0003), but TG and TG-flox did not differ (adjusted *P* = 0.1872). *n* ≥ 13 per group. (**C** and **D**) Representative Western blot (**C**) and quantification (**D**) of spinal cord lysates probed for endogenous LMNB1, exogenous FLAG-tagged human LMNB1 (hLMNB1), β-ACTIN, and GAPDH. hLMNB1 migrates slightly higher than endogenous mouse LMNB1. hLMNB1 levels were normalized to GAPDH and further normalized to Tg-flox. *n* = 3 per group. (**E**) Quantification of spinal cord h*LMNB1* mRNA. h*LMNB1* expression was normalized to *β-Actin* (*Actb*) and further normalized to Tg-flox. *n* = 4 per group. Statistical tests: (**A**) Kruskal-Wallis test with Dunn’s post-hoc test; (**B**) log-rank (Mantel-Cox) test with manual multiple comparison corrections when appropriate; and (**D** and **E**) 1-way ANOVA test with Tukey’s post hoc test. Data are shown as mean ± SEM. *P < 0.05, **P < 0.01, ****P < 0.0001; nonsignificant comparisons are not shown.

Both the TG and TG-flox lines displayed similar hLMNB1 expression levels (Supplemental Figure 1, B and C). Likewise, they showed similar rotarod performance at 7 months of age, which was significantly impaired compared with WT mice, and a reduced lifespan, with ADLD mice succumbing to the disease by 15 months of age (Figure 1, A and B).

To evaluate the efficiency of Cre-mediated recombination and the resulting reduction of hLMNB1, TG-flox mice were crossed with a *Plp1*-cre/ER^T^ mouse line that targets recombination to mature oligodendrocytes in the CNS (27). The resulting bitransgenic offspring (TG-flox;Cre) were then injected with tamoxifen (TAM) to induce ablation. TG-flox;Cre mice showed >75% reduction in both hLMNB1 protein and mRNA expression in the spinal cord relative to that of TG-flox mice (Figure 1, C–E). Consistent with this result, PCR of spinal cord gDNA from the TG-flox;Cre mice confirmed excision of the floxed allele (Supplemental Figure 1, A and D). The spinal cord was selected, as our previous work demonstrated that it exhibits the greatest hLMNB1 overexpression and demyelination pathology in the CNS (19).

High-magnification immunofluorescence images of TG-flox spinal cord sections confirmed that mouse LMNB1 and hLMNB1 (FLAG) colocalized along the nuclear periphery of oligodendrocytes (Supplemental Figure 2A). Further spinal cord immunofluorescence staining demonstrated that hLMNB1 (FLAG) expression was restricted specifically to mature oligodendrocytes, with expression absent in both neurons and astrocytes (Supplemental Figure 2, B and C). hLMNB1 (FLAG) expression was then shown to be significantly reduced by Cre-mediated ablation in TG-flox;Cre mice (Supplemental Figure 2, B–D).

These results demonstrate that TG-flox mice recapitulate the previously generated TG mouse model and human ADLD disease phenotype. Additionally, crossing TG-flox mice with the *Plp1*-cre/ER^T^ line and injecting the offspring with TAM enables a temporally controlled and cell-type-specific ablation of hLMNB1 expression.

### Ablation of hLMNB1 overexpression leads to age-dependent improvements in behavioral outcomes and survival.

Having established an efficient system of hLMNB1 reduction in TG-flox;Cre mice, we next sought to determine if the loss of hLMNB1 overexpression during adulthood and the timing of this ablation influenced disease progression. TAM injections were administered to 2 cohorts of WT, TG-flox, and TG-flox;Cre mice, with injections occurring before the onset of symptoms at 3 months [TG-flox;Cre (3M)] or after onset of symptoms at 6 months of age. [TG-flox;Cre (6M)].

**Figure 2:**
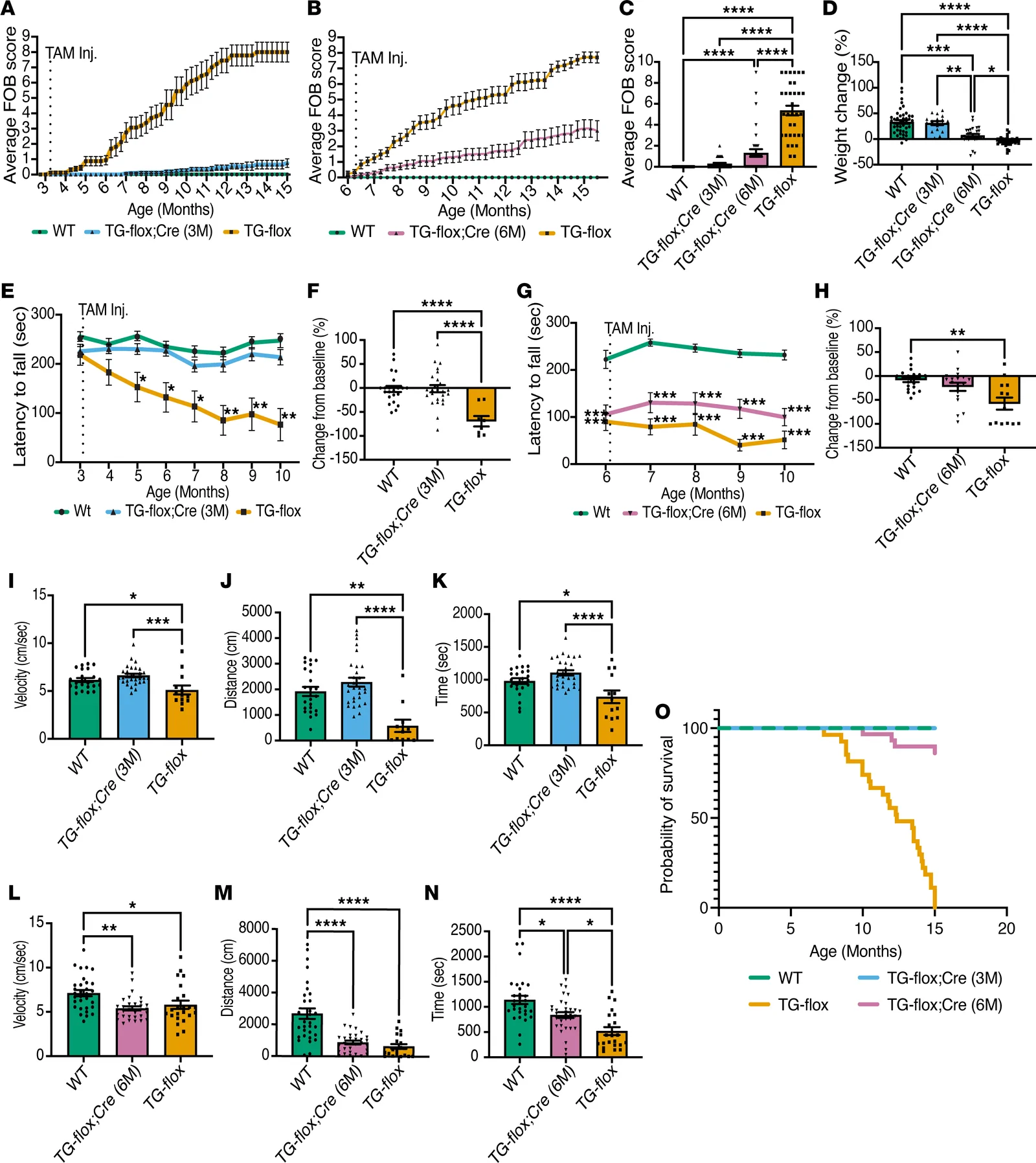
Reduction of hLMNB1 improves motor function in Tg-flox;Cre (3M) and survival in Tg-flox;Cre (3M) and Tg-flox;Cre (6M) mice. (**A** and **B**) Functional observational behavior (FOB) scores at 15 months of age for Tg-flox;Cre (3M) (**A**) and Tg-flox;Cre (6M) (**B**) cohorts. The time point of tamoxifen (TAM) injections is noted. Higher scores indicate more severe impairment. *n* ≥ 19 per group (**A**), *n* ≥ 28 per group (**B**). (**C**) Comparison of FOB scores at 10 months of age among all groups. *n* ≥ 29 for all groups. (**D**) Body weight change from baseline to 10 months of age among all groups. *n* ≥ 17 per group. (**E**–**H**) Monthly rotarod performance and change from baseline to 10 months of age for Tg-flox;Cre (3M) (**E** and **F**) and Tg-flox;Cre (6M) (**G** and **H**) cohorts. The time point of TAM injections is noted. Asterisks depict significance compared with WT in monthly rotarod assessments. *n* ≥ 14 per group (**E**), *n* ≥ 8 per group (**F**), *n* ≥ 15 per group (**G**), *n* ≥ 13 per group (**H**). (**I**–**N**) Open-field analysis at 10 months of age for Tg-flox;Cre (3M) (**I**–**K**) and Tg-flox;Cre (6M) (**L**–**N**) cohorts comparing average ambulatory velocity, total distance traveled, and total movement time. *n* ≥ 13 per group (**I**–**K**); *n* ≥ 23 per group (**L**–**N**). (**O**) Kaplan-Meier survival curves for all groups. Survival curves differed significantly by log-rank (Mantel-Cox) test (*P* < 0.0001). Pairwise log-rank tests with correction for multiple comparisons showed reduced survival in WT vs. TG-flox (adjusted *P* = 0.0006), TG-flox;Cre (3M) vs. TG-flox (adjusted *P* = 0.0006), and TG-flox;Cre (6M) vs. TG-flox (adjusted *P* = 0.0006). Reduced survival was not found in comparisons between WT vs. TG-flox;Cre (3M) (adjusted *P* > 0.9999), WT vs. TG-flox;Cre (6M) (adjusted *P* = 0.0552), and TG-flox;Cre (3M) vs. TG-flox;Cre (6M) (adjusted *P* = 0.3528). *n* ≥ 28 per group. Statistical tests: (**C**, **D**, **J**, and **L**–**N**) Kruskal-Wallis test with Dunn’s post hoc test; (**E** and **G**) mixed effects test with Tukey’s post hoc test; (**H**) Welch’s ANOVA test with Dunnett’s T3 post hoc test; (**F**, **I**, and **K**) 1-way ANOVA test with Tukey’s post hoc test; and (**O**) log-rank (Mantel-Cox) test. Data are shown as mean ± SEM. *P < 0.05, **P < 0.01, ***P < 0.001, ***P < 0.0001; nonsignificant comparisons are not shown.

Cohorts were assessed weekly using a functional observation battery (FOB) score (Supplemental Table 1). TG-flox mice in both cohorts began exhibiting increased FOB scores around 6 months of age, whereas WT mice showed no change. TG-flox;Cre (3M) showed little to no increase in FOB scores, while TG-flox;Cre (6M) mice showed elevated FOB scores compared with those of WT mice, but they were lower than those of TG-flox mice (Figure 2, A and B). At 10 months of age, all groups differed significantly from the TG-flox group by FOB score, with TG-flox;Cre (6M) mice additionally differing from WT mice (Figure 2C). Assessment of weight change from baseline at 10 months of age showed that WT and TG-flox;Cre (3M) mice had similar levels of weight gain, which differed significantly from those of both TG-flox and TG-flox;Cre (6M) mice. TG-flox mice showed weight loss, whereas TG-flox;Cre (6M) mice showed minimal weight gain (Figure 2D).

Monthly rotarod testing was used to assess age-dependent motor dysfunction. In the 3-month cohort, all groups began with a similar performance. WT and TG-flox;Cre (3M) mice remained consistently similar for the testing period, while the TG-flox mice had significantly decreased performance beginning at 5 months of age and worsened with each month (Figure 2E). The change in rotarod performance from 3 to 10 months of age, revealed a minimal decline for both WT and TG-flox;Cre (3M) mice, while TG-flox mice exhibited a significantly greater decrease (Figure 2F). In the 6-month cohort, the TG-flox;Cre (6M) and TG-flox mice initially started with a worse performance compared with that of WT mice, and that was maintained throughout testing (Figure 2G). Notably, when assessing change from baseline, TG-flox;Cre (6M) mice were not significantly different from either WT or TG-flox mice, instead falling between the two (Figure 2H).

At 10 months of age, open-field testing revealed that in the 3-month cohort, WT and TG-flox;Cre (3M) mice had similar average ambulatory velocity, total distance traveled, and total movement time. In contrast, TG-flox mice showed significant deficits in all measures (Figure 2, I–K). In the 6-month cohort, the WT group performed significantly better than both the TG-flox;Cre (6M) and TG-flox groups, which were comparable in average ambulatory velocity and total distance traveled but differed significantly in total movement time (Figure 2, L–N).

Cohorts were followed to approximately 15 months of age, corresponding to the lifespan of TG-flox mice, and survival was assessed. By 15 months of age, no deaths of WT or TG-flox;Cre (3M) mice occurred. TG-flox mice began dying at around 8 months of age, and all were deceased by 15 months. Additionally, TG-flox;Cre (6M) mice showed minimal mortality, which began around 10 months. Paired log-rank (Mantel-Cox) tests revealed no significant differences between WT and TG-flox;Cre (3M) or TG-flox;Cre (6M) mice, but all 3 groups differed significantly from TG-flox mice (Figure 2O).

These results demonstrate that hLMNB1 reduction before the onset of symptoms resulted in the greatest improvement in motor function and survival, with TG-flox;Cre (3M) mice being nearly indistinguishable from WT mice. In contrast, ablation after symptom onset improved FOB scores and survival but provided minimal benefit for motor function.

### Early hLMNB1 reduction prevents spinal cord pathology, while late intervention shows mixed improvement.

**Figure 3:**
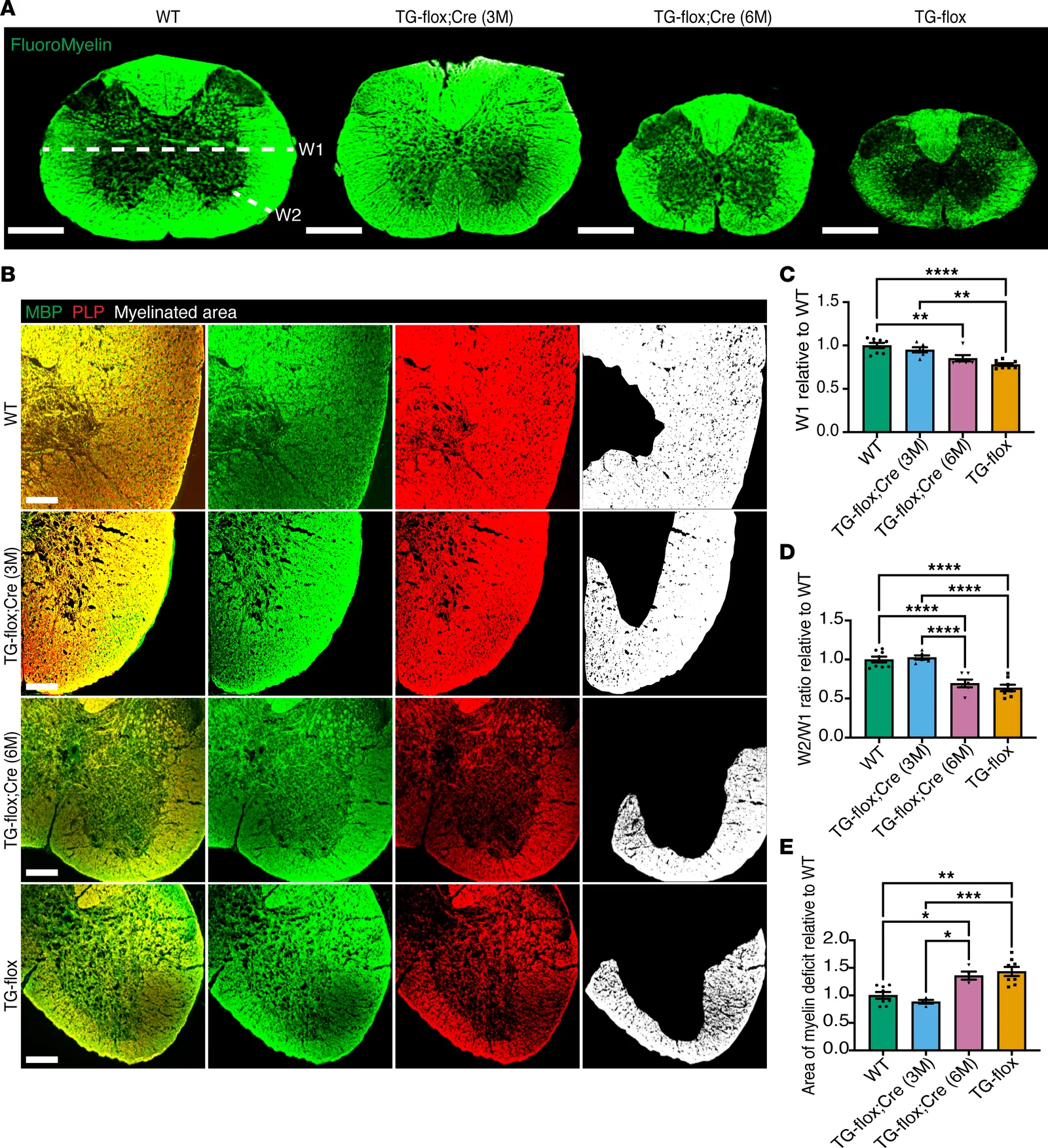
Reduction of hLMNB1 in Tg-flox;Cre (3M) leads to protection from spinal cord shrinkage and white matter deficiency. (**A**) Representative cervical spinal cord sections stained with FluoroMyelin. W1 indicates the total width of the spinal cord; W2 indicates width of the white matter. Scale bar: 500 μm. (**B**) Representative immunofluorescence spinal cord staining for MBP (second column), PLP (third column) overlay of MBP and PLP (first column). The masked area or myelinated area (fourth column) was created artificially with ImageJ software and represent regions of MBP and PLP colocalization. MBP and PLP are myelin proteins. Scale bar: 200 μm. (**C** and **D**) Quantification of the relative spinal cord width (**C**) and relative ratio of white matter width to spinal cord width (**D**), normalized to WT. *n* ≥ 6 per group. (**E**) Quantification of the relative percentage of white matter area with myelin deficiency based on the myelinated masked area images shown in **B**. Group averages were normalized to WT. *n* ≥ 4 per group. Statistical tests: (**C** and **D**) 1-way ANOVA test with Tukey’s post hoc test; (**E**) Welch’s ANOVA test with Dunnett’s T3 post hoc test. Data are shown as mean ± SEM. *P < 0.05, **P < 0.01, ***P < 0.001, ****P < 0.0001; nonsignificant comparisons are not shown).

We next examined the effects of hLMNB1 reduction on spinal cord morphology and white matter integrity using sections stained with FluoroMyelin or myelin basic protein (MBP) and proteolipid protein (PLP) (Figure 3, A and B). Analysis of overall spinal cord width in FluoroMyelin-stained sections revealed that TG-flox and TG-flox;Cre (6M) spinal cords were significantly smaller than those in both WT and TG-flox;Cre (3M) groups (Figure 3C). When the ratio of white matter width to total spinal cord width was quantified, TG-flox;Cre (3M) mice maintained a width ratio similar to that of WT mice, while both TG-flox;Cre (6M) and TG-flox mice had a marked reduction (Figure 3D). Consistent with this, the staining of the spinal cord myelin with MBP and PLP antibodies revealed increased areas of myelin deficiency in the white matter of TG-flox;Cre (6M) and TG-flox mice but not in TG-flox;Cre (3M) mice, compared with WT mice (Figure 3E).

**Figure 4:**
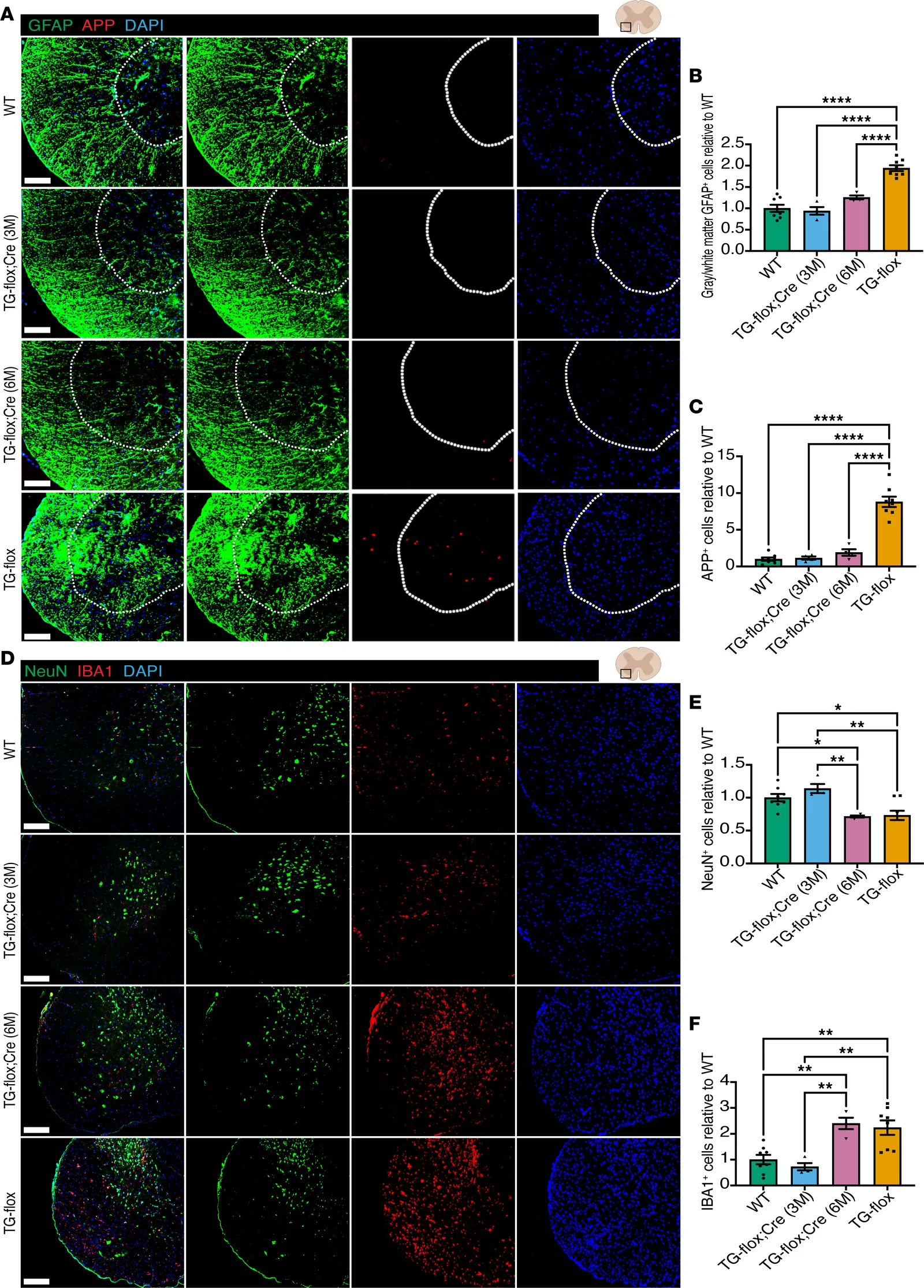
Reduction of hLMNB1 reduces proinflammatory signaling in Tg-flox;Cre (3M) and produces a mixed response in Tg-flox;Cre (6M) mice. (**A**) Representative cervical spinal cord sections stained with GFAP, APP, and DAPI with the boundary between white and gray matter represented by a dotted line. The schematic indicates the region imaged. GFAP labels astrocytes, and APP labels amyloid precursor proteins. Scale bar: 200 μm. (**B** and **C**) Quantification of the ratio of GFAP^+^ cells in gray to white matter (**B**) and total APP^+^ cells (**C**) relative to WT. Each point represents the average of 2 independent fields per mouse. *n* ≥ 4 per group. (**D**) Representative spinal cord sections stained with NeuN, IBA1, and DAPI. The schematic indicates the region imaged. NeuN labels neurons, and IBA1 labels microglia. Scale bar: 200 μm. (**E** and **F**) Quantification of total NeuN^+^ cells (**E**) and IBA1^+^ cells (**F**) relative to WT. Each point represents the average of at least 2 independent fields per mouse. *n* ≥ 4 per group. Statistical tests: (**B**, **E**, and **F**) 1-way ANOVA test with Tukey’s post hoc test; (**C**) Welch’s ANOVA test with Dunnett’s T3 post hoc test. Data are shown as mean ± SEM. *P < 0.05, **P < 0.01, ****P < 0.0001; nonsignificant comparisons are not shown.

To better characterize spinal cord pathology, we performed immunofluorescence staining for astrocytosis and microgliosis. TG-flox mice showed significant signs of inflammation and axonal damage, with elevated GFAP infiltration into the gray matter and increased numbers of APP^+^ cells, while TG-flox;Cre (3M) and TG-flox;Cre (6M) mice maintained levels comparable to WT mice (Figure 4, A–C). In addition, TG-flox;Cre (6M) and TG-flox mice both showed fewer neurons and more microglia, as indicated by NeuN and IBA1 staining, respectively (Figure 4, D–F).

Overall, TG-flox;Cre (3M) mice exhibited no disease-associated pathology compared with TG-flox mice. TG-flox;Cre (6M) mice displayed a mixed phenotype, resembling that of TG-flox mice across all measures, except for APP^+^ cell counts and GFAP infiltration into the gray matter, which were similar to those of WT mice.

### hLMNB1 reduction leads to a decrease in axon abnormalities.

**Figure 5:**
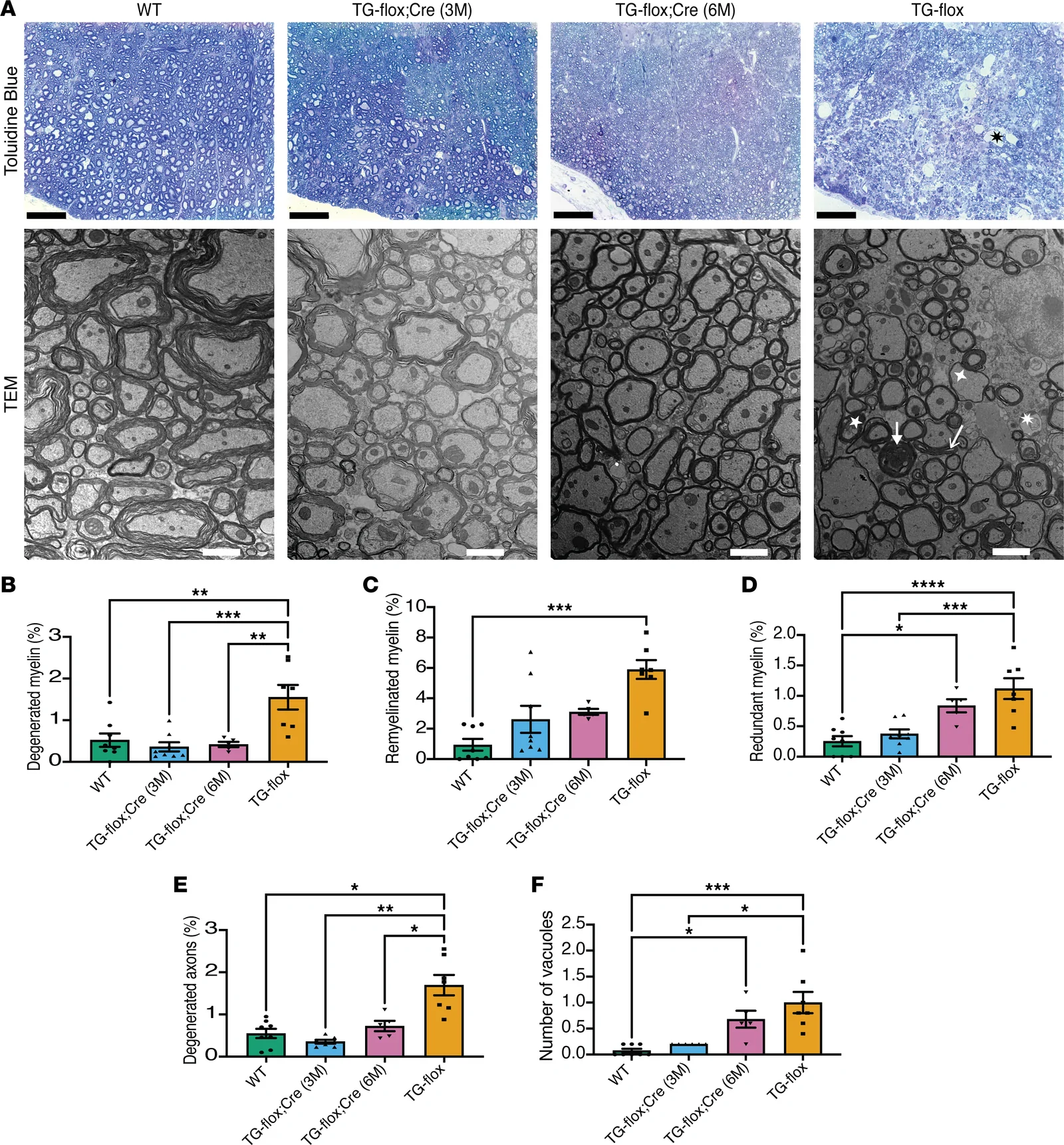
Reduction of hLMNB1 decreases axon abnormalities for both Tg-flox;Cre (3M) and Tg-flox;Cre (6M) mice. (**A**) Representative spinal cord sections stained with toluidine blue or imaged by transmission electron microscopy (TEM). Symbols indicate examples of axonal or myelin abnormalities: thick arrow = degenerated myelin, 4-point star = remyelinated myelin, thin arrow = redundant myelin, 5-point star = degenerated axon, 7-point star = vacuole. Scale bar: 50 μm (toluidine blue); 2,000 nm (TEM). (**B**–**F**) Quantification of the percentage of degenerated myelin (**B**), remyelinated myelin (**C**), redundant myelin (**D**)**,** degenerated axons (**E**), and the number of vacuoles (**F**). Each point represents the average of 5 independent fields per mouse. *n* ≥ 5 per group. Statistical tests: (**B** and **D**) 1-way ANOVA test with Tukey’s post hoc test; (**C** and **F**) Kruskal-Wallis test with Dunn’s post hoc test; (**E**) Welch’s ANOVA test with Dunnett’s T3 post hoc test. Data are shown as mean ± SEM. *P < 0.05, **P < 0.01, ***P < 0.001, ***P < 0.0001; nonsignificant comparisons are not shown.

Consistent with the immunofluorescence data, TG-flox mice exhibited pronounced axonal pathology, including loss of axon organization and increased vacuolation, as observed in both toluidine blue staining and TEM (Figure 5A). Relative to WT mice, TG-flox mice displayed significantly higher percentages of degenerated myelin, remyelinated myelin, redundant myelin, degenerated axons, and increased vacuole counts. Both TG-flox;Cre (3M) and TG-flox;Cre (6M) cohorts showed marked improvement. TG-flox;Cre (3M) mice were largely comparable to WT mice, except for a nonsignificant increase in remyelinated myelin, which was similarly increased in TG-flox;Cre (6M) mice. TG-flox;Cre (6M) mice were also generally similar to WT mice, although redundant myelin and vacuole numbers remained closer to levels in TG-flox mice (Figure 5, B–F).

To further characterize axonal differences, we used AxonDeepSeg, a deep learning tool for axon segmentation (28). This revealed that the mean g-ratio, defined as the ratio of the inner axonal diameter to the total outer diameter of the myelinated fiber, was significantly higher in both TG-flox;Cre (6M) and TG-flox mice than in WT mice. TG-flox;Cre (3M) mice exhibited a mean g-ratio comparable to that of WT mice, and both means were significantly lower than TG-flox mice (Supplemental Figure 3A). When plotting g-ratio against axon diameter using total axon counts, TG-flox;Cre (3M), TG-flox;Cre (6M), and TG-flox axons showed a tendency toward higher g-ratios at larger diameters, with visually steeper slopes compared with WT axons, indicating thinner myelin sheaths around larger-diameter axons (Supplemental Figure 3B). Consistent with this observation, comparison of g-ratios across axon diameter bins revealed a statistically significant increase in TG-flox;Cre (3M), TG-flox;Cre (6M), and TG-flox g-ratios compared with WT g-ratios, beginning with axons 1.0–1.5 μm and continuing to >3 μm (Supplemental Figure 3C).

Regardless of the timing of TAM injection, reduction of hLMNB1 improved axonal organization, health, and g-ratio, with TG-flox;Cre (3M) mice showing the greatest improvement toward WT levels. However, further AxonDeepSeg analysis of g-ratio, based on axon diameter bins, revealed a pronounced difference between WT and Tg-flox;Cre (3M) mice that became more evident at larger axon diameters.

### Reduction of hLMNB1 overexpression yields mixed improvement of ADLD disease progression biochemical markers.

**Figure 6:**
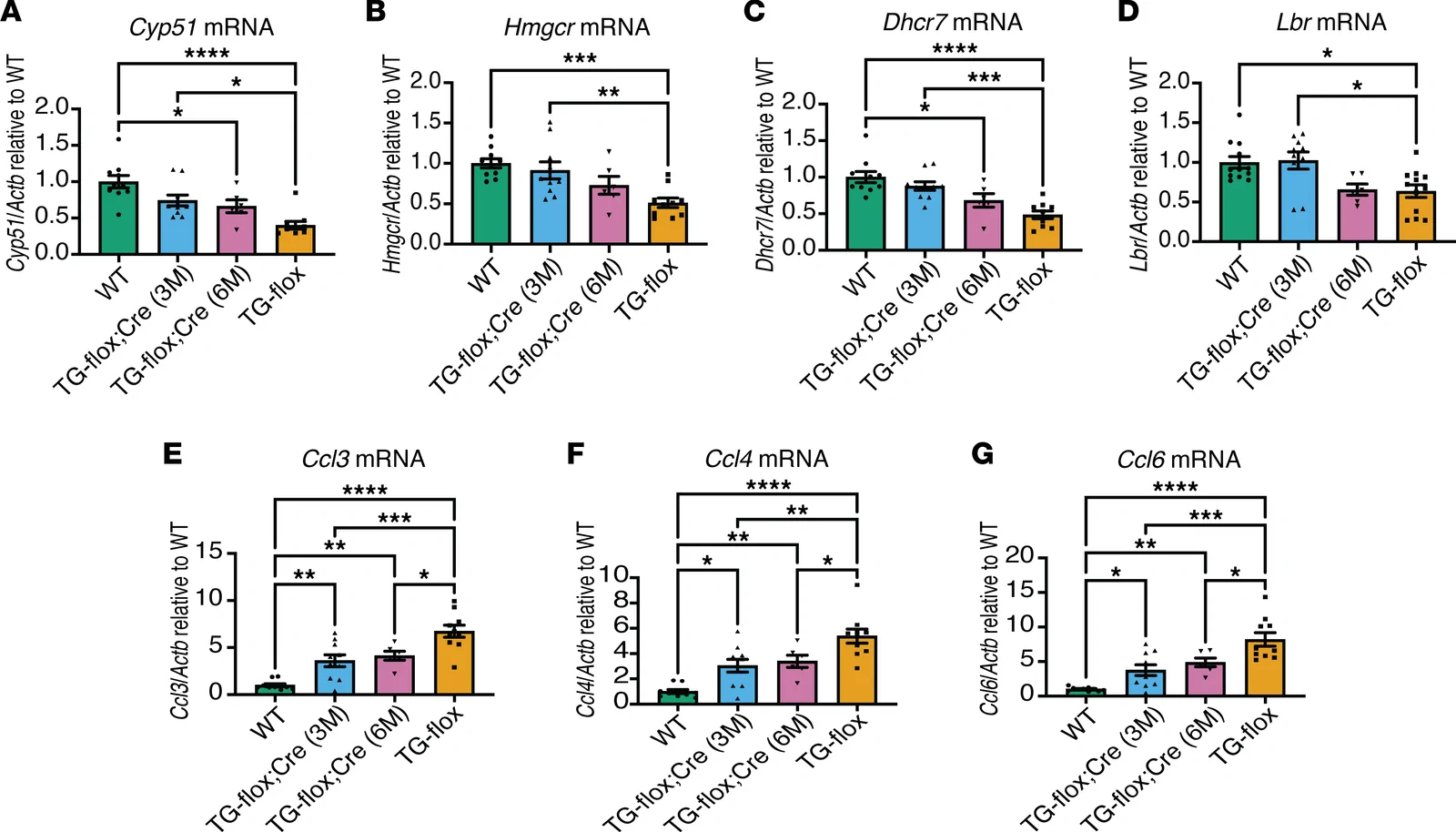
Reduction of hLMNB1 restores lipid synthesis gene expression to WT levels in Tg-flox;Cre (3M) mice and decreases the elevation of cytokine gene expression in both Tg-flox;Cre (3M) and Tg-flox;Cre (6M). Real-time qPCR analysis of lipid synthesis genes, *Cyp51* (**A**), *Hmgcr* (**B**), *Dhcr7* (**C**), and *Lbr* (**D**), and proinflammatory cytokine genes, *Ccl3* (**E**), *Ccl4* (**F**), *Ccl6* (**G**). mRNA expression levels were normalized to *β-actin* (*Actb*) and further normalized to WT. *n* ≥ 6 per group. Statistical tests: 1-way ANOVA test with Tukey’s post hoc test. Data are shown as mean ± SEM. *P < 0.05, **P < 0.01, ***P < 0.001, ****P < 0.0001; nonsignificant comparisons are not shown).

Our prior work with the TG mouse model has demonstrated that reductions in the expression of genes involved in lipid synthesis and increases in proinflammatory cytokine markers were the most characteristic biochemical alterations accompanying disease progression (19). We analyzed the expression of representative lipid-synthesis genes and observed similar reductions, with TG-flox mice showing significant reductions in *Cyp51*, *Hmgcr*, *Dhcr7*, and *Lbr* expression. TG-flox;Cre (3M) mice remained comparable to WT mice and differed significantly from TG-flox mice. TG-flox;Cre (6M) mice did not differ significantly from TG-flox mice and showed a significant reduction relative to WT mice in *Cyp51* and *Dhcr7* expression (Figure 6, A–D). Consistent with these findings, immunofluorescent staining and analysis of mature oligodendrocytes, represented by CC1 staining, revealed a significant reduction in FASN intensity in all TG mice relative to WT mice, with the greatest reductions in TG-flox;Cre (6M) and TG-flox groups (Supplemental Figure 4, A and B).

We next assessed representative cytokine gene expression, which revealed that TG-flox mice demonstrated an approximately 5-fold increase in *Ccl3*, *Ccl4*, and *Ccl6* compared with WT mice. Unexpectedly, both TG-flox;Cre (3M) and TG-flox;Cre (6M) mice demonstrated similar cytokine expression levels that were intermediate to, and significantly different from, those observed in WT and TG-flox mice (Figure 6, E–G).

Thus, lipid synthesis gene expression improved most in the TG-flox;Cre (3M) mice, and the timing of hLMNB1 overexpression ablation did not alter proinflammatory gene expression. These findings suggest that hLMNB1 overexpression may directly drive a reduction in lipid synthesis but may not independently maintain the increased proinflammatory response.

### hLMNB1 reduction normalizes neurodegeneration-associated biomarker levels.

We next measured the protein concentrations of pTau181, Aβ40, Aβ42, GFAP, and NFL in mouse spinal cords to determine whether these biomarkers that have been previously shown to track disease progression and severity in other neurodegenerative disorders might also be relevant to ADLD (29–33).

**Figure 7:**
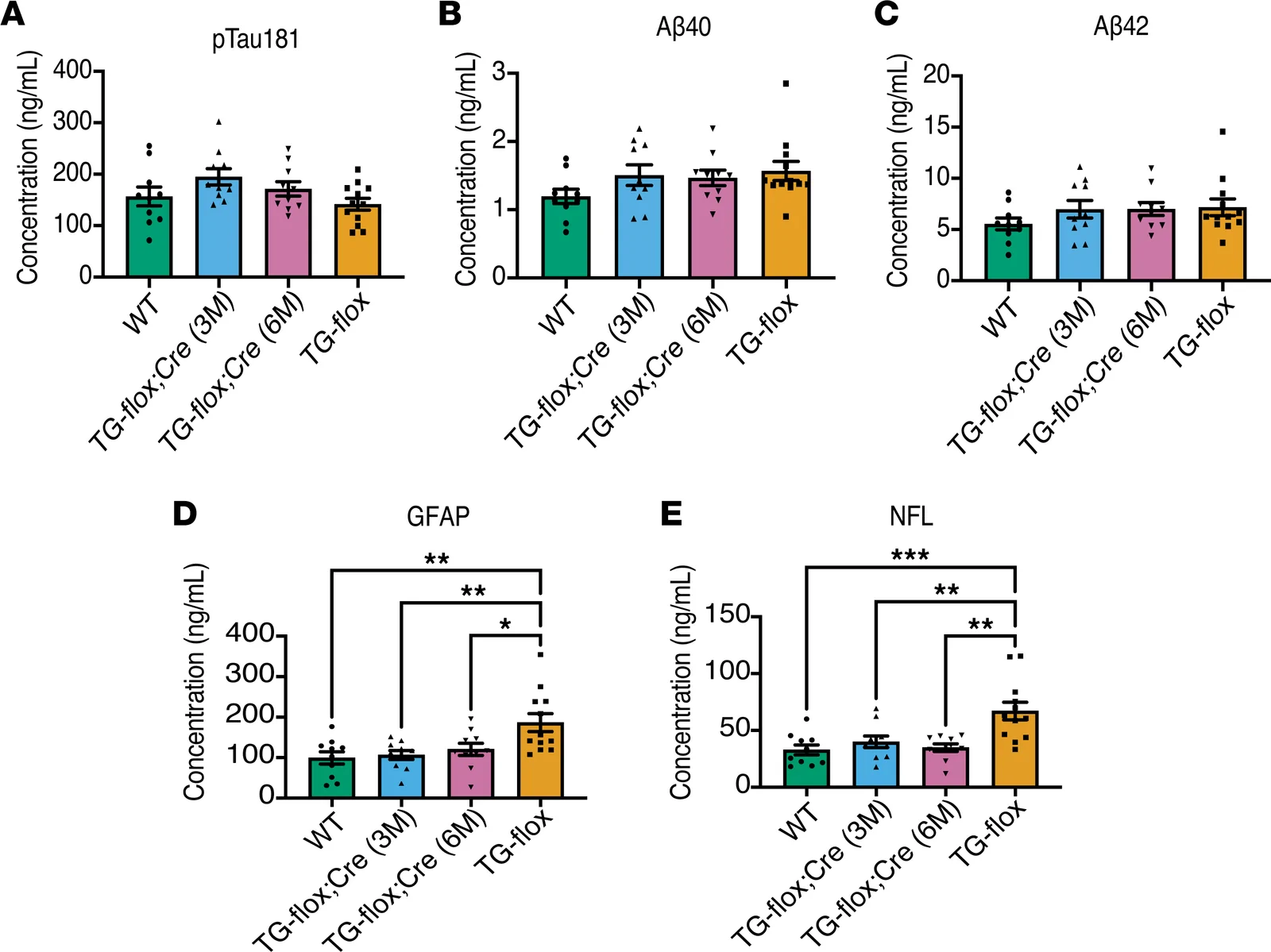
Reduction of hLMNB1 decreases the concentration of neurodegeneration-associated biomarkers in both Tg-flox;Cre (3M) and Tg-flox;Cre (6M) mice. Quantification of neurodegeneration biomarkers in spinal cord lysates: pTau181 (**A**), Aβ40 (**B**), Aβ42 (**C**), GFAP (**D**), and NFL (**E**). *n* ≥ 10 per group. Statistical tests: (**A**–**E**) 1-way ANOVA test with Tukey’s post hoc test. Data are shown as mean ± SEM. *P < 0.05, **P < 0.01, ***P < 0.001; nonsignificant comparisons are not shown.

Concentrations of pTau181, Aβ40, and Aβ42 were found to be similar across all groups (Figure 7, A–C), suggesting that these pathogenic proteins are not prominently altered in this model. In contrast, GFAP and NFL concentrations were significantly higher in the TG-flox group compared with the other groups, highlighting the disease’s axonal damage and astrocytic activation. Interestingly, WT, TG-flox;Cre (3M), and TG-flox;Cre (6M) mice exhibited similar GFAP and NFL levels (Figure 7, D and E).

### Proinflammatory, but not lipid synthesis, gene expression correlates with hLMNB1 reduction and motor function.

We next performed exploratory correlation analyses to examine whether *hLMNB1* expression was associated with pathogenic biomolecular changes, including decreased lipid synthesis and increased proinflammatory gene expression. We also tested whether *hLMNB1* levels and these biomolecular markers were correlated to rotarod performance at 10 months of age as a measure of disease severity. Analyses were performed on the combined genotype dataset (global), with TG-flox;Cre (3M) and TG-flox;Cre (6M) grouped as TG-flox;Cre.

Globally, *hLMNB1* expression was negatively correlated with rotarod performance (Supplemental Figure 5A). Lipid synthesis gene expression (*Cyp51*, *Hmgcr*, *Dhcr7*, and *Lbr*) decreased as *hLMNB1* expression increased across the dataset but was not significantly associated with rotarod performance (Supplemental Figure 5, B and C). Proinflammatory cytokine expression (*Ccl3*, *Ccl4*, and *Ccl6*) was positively associated with *hLMNB1* levels and negatively correlated with rotarod performance (Supplemental Figure 5, D and E).

Together, these findings indicate that *hLMNB1* expression is associated with key pathogenic molecular changes in the ADLD mouse model, particularly alterations in lipid synthesis and proinflammatory gene expression. In addition, the expression of the proinflammatory cytokines *Ccl3*, *Ccl4*, and *Ccl6* was strongly associated with both *hLMNB1* levels and motor performance, suggesting their potential utility as biomarkers for disease severity and therapeutic response.

### Lmnb1 knockout in adult mice does not impact motor function, survival, or gross CNS organization.

Having demonstrated the benefits of exogenous hLMNB1 reduction in the ADLD mouse model, we next sought to determine if reducing endogenous LMNB1 had deleterious effects in adult mice. This is a critical consideration for any LMNB1-reducing therapy, which would likely be administered only during adulthood. Previous research has shown that an embryonic knockout of *Lmnb1* in the forebrain is lethal, with mice dying soon after birth with a reduced brain size and abnormal cortical neuron layering (Supplemental Figure 6, A and B) (16).

**Figure 8:**
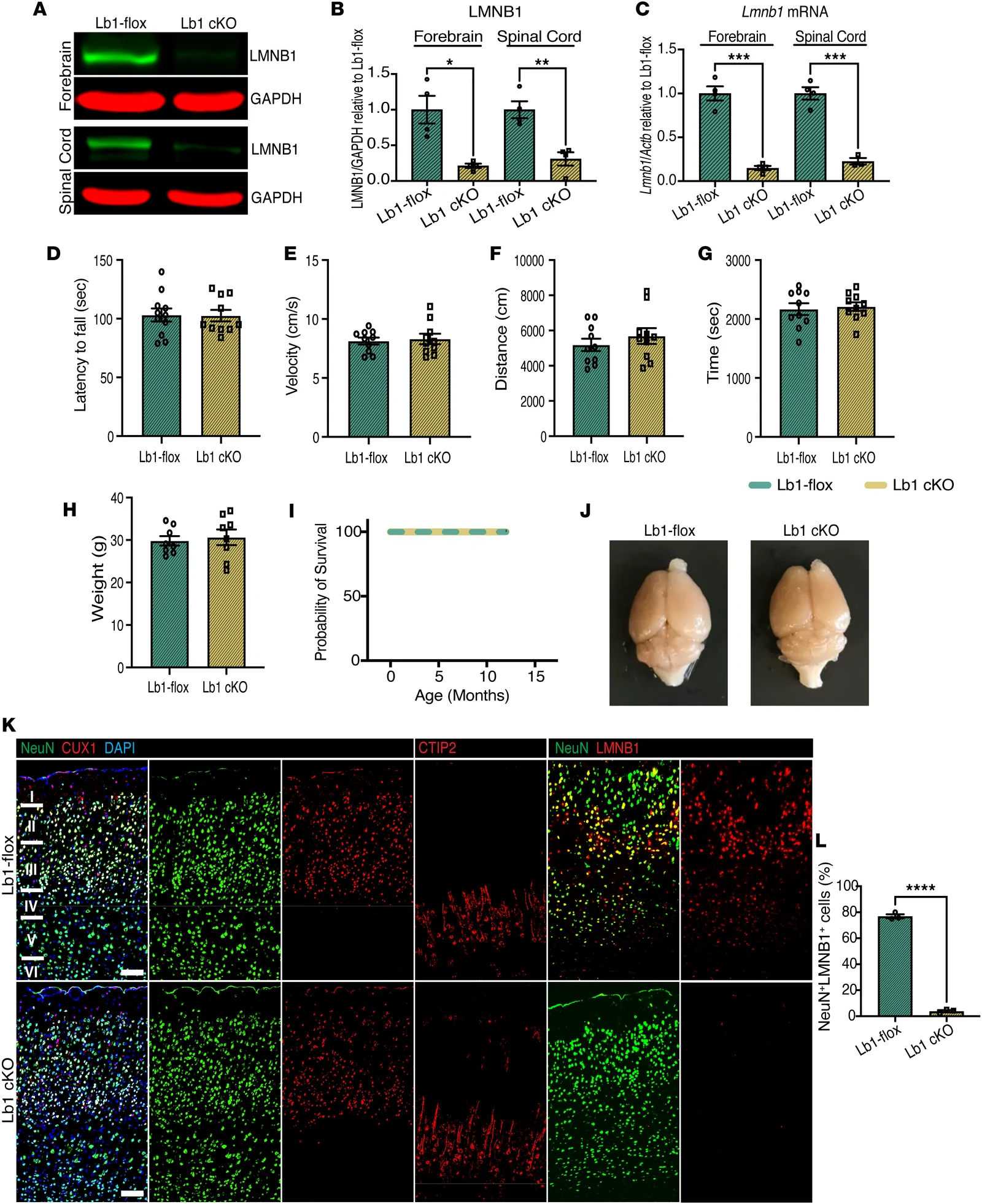
Ubiquitous knockout of *Lmnb1* in adult mice does not impair motor function, survival, or cortical neuron layering. (**A** and **B**) Representative Western blot (**A**) and quantification (**B**) of forebrain and spinal cord lysates probed for endogenous murine LMNB1 and GAPDH. LMNB1 levels were normalized to GAPDH and further normalized to Lb1-flox. *n* = 3 per group. (**C**) Quantification of forebrain and spinal cord *Lmnb1* mRNA expression. *Lmnb1* levels were normalized to *β-actin* (*Actb*) and further normalized to Lb1-flox. *n* = 4 per group. (**D**) Rotarod performance at 10 months of age. *n* ≥ 10 per group. (**E**–**G**) Open-field analysis at 10 months of age comparing average ambulatory velocity (**E**), total distance traveled (**F**), and total movement time (**G**). *n* = 10 per group. (**H**) Body weight at 12 months of age. *n* = 8 per group. (**I**) Kaplan-Meier survival curves to 12 months of age. *n* = 8 per group. No significance by log-rank (Mantel-Cox) test (*P* > 0.9999). (**J**) Representative forebrain images taken at 12 months of age. (**K**) Representative cortical sections stained with a combination of NeuN, CUX1, DAPI, CTIP2, or LMNB1 antibodies. NeuN labels neurons, CUX1 labels layers II to upper IV, and CTIP2 labels lower IV to VI. Cortical neuron layers are labeled by roman numerals in Lb1-flox images. Scale bar: 50 μm. (**L**) Quantification of the percentage of NeuN^+^ cells that were also LMNB1^+^. *n* = 3 per group. Each point represents 1 independent field per mouse. Statistical tests: (**B**–**H** and **L**) Welch’s *t* test; (**I**) log-rank (Mantel-Cox) test. Data are shown as mean ± SEM. *P < 0.05, **P < 0.01, ***P < 0.001, ****P < 0.0001 nonsignificant comparisons are not shown.

To determine if adult knockout of *Lmnb1* carries detrimental effects, we crossed *Lmnb1*-flox/flox mice with a UBC-Cre-ER^T2^ strain (34). Resulting *Lmnb1*-flox/flox (Lb1-flox) and *Lmnb1*-flox/flox; UBC-Cre-ERT2 (Lb1 cKO) mice were injected with TAM at 3 months of age to induce ubiquitous *Lmnb1* knockout. Lb1 cKO mice demonstrated an almost complete loss of LMNB1 protein and *Lmnb1* mRNA expression in the forebrain and spinal cord (Figure 8, A–C). At 10 months of age, Lb1 cKO mice showed no impairment in motor function, as assessed by rotarod and open field measurements (average velocity, total distance traveled, and total movement time) (Figure 8, D–G). At 12 months of age, Lb1 cKO body weight and survival were similar to those in Lb1-flox mice (Figure 8, H and I). Additionally, no alteration in brain size was observed in Lb1 cKO mice, and cortical neuron layering, as assessed by NeuN, CUX1, and CTIP2 staining, remained normal despite a near absence of LMNB1 in neurons (Figure 8, J–L, and Supplemental Figure 6, C and D).

The results indicate that a ubiquitous knockout of *Lmnb1* in adult mice does not cause any overt CNS phenotype. While we cannot rule out more subtle alterations in the CNS and other systems, Lb1 cKO mice were indistinguishable from Lb1-flox mice across all measures analyzed, suggesting that *Lmnb1* is not essential for survival in the adult mouse or that compensatory mechanisms after development can maintain function. Importantly, these findings can address potential safety issues associated with targeting LMNB1 reduction in adults using gene therapy or other approaches.

## Discussion

Our work demonstrates that reducing LMNB1 in an in vivo model of the disease has therapeutic efficacy. The TG-flox mice exhibit features reminiscent of ADLD, including age-related motor dysfunction, decreased survival, and demyelination. They also show decreased lipid synthesis and an elevated proinflammatory response, consistent with our previously generated ADLD mouse line (19). In contrast, reduction of hLMNB1 mitigates or prevents disease outcomes, with many disease-associated phenotypes reduced and, in some cases, restored to WT levels.

Furthermore, our results indicate that the timing of hLMNB1 reduction is critical for halting or slowing disease progression. When hLMNB1 was reduced before the expected onset of visible symptoms, TG mice showed improvements in motor function, survival, white matter integrity, myelination, lipid-synthesis gene expression, and proinflammatory signaling, largely comparable to WT. In contrast, reduction of hLMNB1 after symptom onset resulted in modest improvements, with the largest benefit being an increase in survival (Supplemental Figure 7). These data indicate that lowering hLMNB1 overexpression in our mouse model of ADLD can halt or slow disease progression and that early intervention offers the greatest therapeutic benefit.

Despite the benefits of hLMNB1 reduction, some subtle disease pathology persisted, even in the TG-flox;Cre (3M) mice, with more severe pathology in the late-intervention cohort [TG-flox;Cre (6M)]. Both early- and late-intervention cohorts similarly exhibited elevated proinflammatory mRNA expression and increased g-ratios at larger axon diameters than in WT mice. Although proinflammatory expression was reduced relative to that in TG-flox mice, it remained elevated compared with WT mice, suggesting that the neuroinflammatory response is partially, rather than solely, driven by hLMNB1 overexpression. Additionally, the residual pathology observed in the early intervention cohort suggests that pathological changes that occur early in life due to LMNB1 overexpression may persist even after this overexpression is halted. Consistent with the concept of an early-life initiating event, Huntington’s disease, another late adult-onset neurodegenerative disease, has been shown to exhibit abnormal neurodevelopment as early as 13 weeks of gestation (35, 36). These results underscore the importance of identifying and characterizing subtle pathological changes during early life or the prodromal period of ADLD to understand disease progression better and optimize the timing of therapeutic interventions.

Our results also identify potential biomarker candidates that may reflect hLMNB1 levels and disease severity. In our model, spinal cord protein lysates from TG-flox mice showed elevated NFL and GFAP levels, whereas WT and both hLMNB1-reduced cohorts [TG-flox;Cre (3M) and (6M)] exhibited similarly low levels. NFL and GFAP are commonly used neurodegenerative markers and indicate axonal injury and astrocyte activation, respectively (37–39). This would suggest that NFL and GFAP track LMNB1 levels rather than disease severity, as the TG-flox;Cre (3M) and (6M) mice have similarly reduced hLMNB1 levels but different degrees of residual disease phenotypes, with the (6M) mice being much more severely affected compared with the (3M) mice. In contrast, correlation analyses revealed associations between the expression levels of proinflammatory cytokines (*Ccl3*, *Ccl4*, and *Ccl6*) and both *hLMNB1* levels and motor function. Notably, CCL3 and CCL4 have been reported as markers of neuroinflammation in amyotrophic lateral sclerosis, where their levels correlated with disease progression, disease severity, and patient survival (40–43). These results suggest that expression of proinflammatory genes and their corresponding protein concentrations may reflect ADLD disease severity, potentially serving as ADLD biomarkers, and aid in monitoring LMNB1-reducing therapies. However, further validation, including assessment of samples from patients with ADLD, is necessary to determine their reliability.

Previous studies have shown that *Lmnb1* is essential during development, as embryonic forebrain-specific knockout of *Lmnb1* results in perinatal lethality and cortical neuron layer defects (16). Consistent with this developmental requirement, *Lmnb1*-null mice have, likewise, been found to die soon after birth with defects in brain, bone, and lung development (44). In contrast, cell-specific depletion of *Lmnb1* in either keratinocytes or hepatocytes does not cause obvious or detrimental abnormalities, underscoring a strong cell-type dependence of *Lmnb1* function (45, 46). Our work demonstrates that ubiquitous *Lmnb1* knockout in adult mice does not detectably alter motor function, weight, survival, or cortical neuron layering. While more subtle alterations cannot be ruled out and warrant further investigation, these findings contrast sharply with the severe developmental defects and lethality observed in embryonic *Lmnb1* knockout models (16). These results suggest that the roles of LMNB1 during development are distinct from those during adulthood, where its expression may be dispensable or redundant. It will also be important to follow these mice beyond the 12-month endpoint to rule out any late-onset adverse effects, especially since ADLD is slowly progressive and LMNB1-depleting treatments may need to be continued through later ages in patients. Similar to our findings with LMNB1, huntingtin, the gene mutated in Huntington’s disease, is embryonic lethal when completely knocked out during development but leads to no discernible phenotype when knocked out in adulthood (>4 months) (47, 48). Together, these results highlight both the time-specific and cell-specific roles of *Lmnb1* and suggest that reducing LMNB1 levels in adulthood would be well tolerated and therefore a safe therapeutic target for treating ADLD.

It is important to point out that a limitation of our study is that the Cre-Lox system is not directly translatable to patients. Nonetheless, our results demonstrate that hLMNB1 reduction in our ADLD mouse model yields clear beneficial effects, providing a strong impetus to develop translatable approaches for reducing gene expression in humans. These potential strategies include antisense oligonucleotides (ASOs), adeno-associated viral–mediated (AAV-mediated) shRNA, and CRISPR-mediated knockdown. While each approach possesses unique advantages and limitations, the best strategy for ADLD remains to be determined. Our results indicate that oligodendrocytes are the key cell type that must be targeted for ADLD treatment. Therapeutic challenges include targeting LMNB1 reduction specifically to oligodendrocytes and managing potential long-term treatment given the slowly progressive nature of the disease, which might favor strategies requiring a single administration, such as AAV shRNA approaches, over those requiring repeated administration, like ASOs. However, AAV therapies present potential toxicity risks related to immune reactions against capsid proteins, especially if repeated administration is required to achieve sustained LMNB1 reduction. Notably, our Cre-Lox model achieves sustained and irreversible reduction of hLMNB1 overexpression after a single TAM administration regimen, at the specified time point. In patients with ADLD, we would predict that a similar sustained reduction of LMNB1 throughout the lifespan would also be required to achieve therapeutic efficacy. This outcome is much easier to observe in mice because of their shorter lifespan and the relatively rapid progression of the disease. Given the potentially long duration of treatment in humans, achieving this longevity presents a major challenge that future therapeutic strategies must overcome to ultimately develop an effective therapy for ADLD.

In conclusion, we demonstrate, for the first time to our knowledge, proof of concept for the in vivo therapeutic efficacy of reducing LMNB1 overexpression using a potentially novel inducible hLMNB1 ADLD mouse line. Both early- and late- intervention led to improved disease outcomes, with early intervention providing the greatest benefit. Moreover, we showed that the ubiquitous loss of *Lmnb1* expression in adult mice does not produce discernible phenotypes. Collectively, our results highlight the therapeutic potential of LMNB1-reducing approaches for ADLD and provide a rationale for developing translatable strategies to reduce LMNB1.

## Methods

### Sex as a biological variable.

For all experiments, both males and females were used in approximately equal proportions. Sex was not considered a biological variable, as we have not previously observed any sex-based differences in phenotypes.

### Mouse husbandry and generation of experimental mouse lines.

Mice were housed under a 12-hour light/dark cycle with an ambient temperature of 68°F–76°F, a relative humidity of 30% to 70%, and ad libitum access to food and water.

To generate the TG-flox mouse line capable of inducible ablation of hLMNB1 overexpression, the previously described *Plp-FLAG-LMNB1* cassette (19) was modified by site-specific mutagenesis to insert loxP sites flanking the FLAG-hLMNB1 construct. The modified cassette was purified and injected into C57BL/6J mouse embryos by the University of Pittsburgh Transgenic Core. Two founder lines carrying the cassette were identified by PCR and assessed for hLMNB1 expression by Western blot and quantitative real-time qPCR (qRT-PCR). hLMNB1 expression was found to be comparable across the two lines, and one line was used for all subsequent experiments.

TG-flox mice were crossed to the *Plp1*-cre/ER^T^ line (strain no. 005975) (27) from The Jackson Laboratory to generate WT, TG-flox, and TG-flox;Cre offspring. Additionally, the *Lmnb1*-flox (strain no. 032558) (45) and UBC-Cre-ER^T2^ (strain no. 008085) (34) mouse lines from The Jackson Laboratory were crossed to generate Lb1-flox and Lb1 cKO mice. All mouse strains were maintained on a C57BL/6 J background. Genotyping was performed using primers listed in Supplemental Table 2.

Mice were monitored weekly for signs of deterioration and euthanized upon reaching endpoint criteria defined in the mouse behavior assessment section.

### TAM preparation, administration, and hLMNB1 knockout validation.

TAM (Sigma-Aldrich) was dissolved at 20 mg/mL in corn oil (Sigma-Aldrich) following standard protocols from The Jackson Laboratory. Intraperitoneal TAM injections were given to WT, TG-flox, and TG-flox;Cre mice at approximately 3 [TG-flox;Cre (3M)] or 6 [TG-flox;Cre (6M)] months of age or to Lb1-flox and Lb1 cKO mice at around 3 months of age, at a dose of 75 mg TAM/kg of body weight. Injections were performed over 10 days, with a 2-day interval between the fifth and sixth injections.

To test the excision efficacy of the hLMNB1 overexpression cassette, genomic DNA was isolated from the mouse spinal cord using the Gentra Puregene kit (Qiagen) according to the manufacturer’s protocol. Knockout PCR primers were designed to flank the loxP sites, resulting in a smaller band upon successful recombination in Tg-flox;Cre mice (Supplemental Figure 1A). Knockout primers are listed in Supplemental Table 2.

### Mouse behavior assessment.

Mice were weighed biweekly and assessed weekly using a FOB score (Supplemental Table 1), with higher scores indicating more severe disease pathology. An FOB score of 9 was defined as the experimental endpoint, at which point mice were euthanized, and their age at euthanasia was noted for survival analysis. Mice that did not reach this endpoint were euthanized at approximately 15 months of age for the *Plp-FLAG-LMNB1*-flox cohorts and 12 months of age for the *Lmnb1-*flox cohorts.

Rotarod and open-field tests were conducted at the University of Pittsburgh’s Preclinical Phenotyping Core, following previously described protocols (49). Briefly, the rotarod test consisted of 3 runs of an accelerating protocol, in which the rotation speed rose from 4 to 40 rpm over 300 seconds. Mice that did not fall were assigned a latency of 300 seconds, and performance was analyzed using the average latency to fall across trials. Open-field testing was conducted over 30 minutes, with activity recorded in 5-minute bins; analyses were performed using either the summed total or the average across bins.

### RNA isolation and qRT-PCR.

RNA was isolated from forebrain and cervical spinal cord tissue, and cDNA was synthesized following previously described protocols (10). Briefly, RNA was extracted using TRIzol reagent (Invitrogen) based on the manufacturer’s protocol. 1 μg RNA was used to synthesize cDNA with the qScript cDNA Synthesis Kit (Quanta Bio). qRT-PCR was performed using PowerUp SYBR Master Mix (Thermo Fisher Scientific) on an ABI QuantStudio 12K Flex (Applied Biosystems). Gene expression analysis was conducted using previously described protocols (10), in which the ΔΔC_T_ method (50) was applied, with *β-actin* mRNA serving as the endogenous control. Real-time qPCR primers are listed in Supplemental Table 2.

### Protein isolation, Western blotting, and biomarker analysis.

Protein from mouse forebrain and cervical spinal cord tissue was isolated and blotted as previously described (19). Briefly, tissues were homogenized in a mixture of T-PER and 1x protease inhibitor cocktail (Fisher Scientific), and Western blotting was carried out with 30 μg of protein per sample loaded on a 10% SDS-polyacrylamide gel. Blots were imaged with the LI-COR Odyssey CLx infrared scanner and analyzed using Image Studio software (LI-COR Biosciences). Antibodies and their dilutions are listed in Supplemental Table 3. Images of uncropped Western blots are in the supplemental materials.

Mouse spinal cord lysates were stored at –80°C until analysis, and proteomic biomarkers were quantified using validated single-molecule array (Simoa) assays on an HD-X instrument (Quanterix) following the manufacturer’s protocol. Aβ40, Aβ42, NFL, and GFAP concentrations were measured using the Neurology 4-Plex E assay (Quanterix, #103670), while p-Tau181 levels were measured with the p-Tau181 V2.1 Advantage Kit (Quanterix,#104111). Quality control samples at 3 different concentrations were analyzed before each run to assess assay reproducibility. Within-run coefficients of variation for the quality control samples were as follows: 2.99% for NFL, 3.95% for GFAP, 13.8% for Aβ42, 1.60% for Aβ40, and 9.59% for p-tau181.

### Immunofluorescence staining.

For histological analysis, mice were anesthetized, and an intracardiac perfusion was performed first with cold 1x PBS and then with cold 4% PFA (Santa Cruz). Tissues were then post-fixed in 4% PFA and cryoprotected by serial sucrose infiltration before being embedded in O.C.T. freezing medium (Fisher Scientific). Cryosections were taken between the spinal cord cervical regions C3 and C6 at 10 μm and stored at –80°C.

Immunofluorescence staining was carried out following standard staining procedures using a blocking solution of 5% normal donkey serum (Fisher Scientific) and 0.2% Tween-20 (Fisher Scientific) in 1x PBS. Primary or secondary antibodies were diluted in a 1x PBS solution with 1% bovine serum albumin (Sigma Aldrich) and 0.2% Tween-20 (Fisher Scientific). Antibodies and their dilutions are listed in Supplemental Table 3. FluoroMyelin staining was performed according to the manufacturer’s instructions (Thermo Fisher Scientific). Images were acquired on a Leica CTR5000 fluorescence microscope or a Nikon Eclipse Ti2 confocal microscope, with identical exposure settings applied across samples. Each slide contained at least one section from every experimental group within a cohort.

Spinal cord and white matter width were measured on FluoroMyelin-stained sections using ImageJ (NIH). A straight line was drawn across the widest portion of each region, and distances were recorded in micrometers using the image scale bar for calibration.

Myelin deficiency was quantified in spinal cord sections costained for MBP and PLP using ImageJ. The white matter region was manually outlined based on the colocalization of MBP and PLP, and the surrounding area was cleared. Images were then converted to 8-bit and thresholded to highlight unstained regions corresponding to myelin-deficient areas. The “Analyze Particles” function was used to calculate these regions as a percentage area relative to the manually defined white matter region.

For all fluorescent staining and quantification, the investigator was blinded to the experimental groups. All analyses were carried out using the average of ≥2 independent fields or ≥16 cells per individual sample.

### Transmission electron microscopy and AxonDeepSeg analysis.

Mouse anesthetization and intracardiac perfusion were performed similarly to the procedures used for immunofluorescence, but with 4% PFA and 2.5% glutaraldehyde in sodium cacodylate buffer. The tissue was then post-fixed in the same solution overnight at room temperature. Postfixed cervical region C3–C6 spinal cord samples were rinsed in 0.1 M sodium cacodylate buffer, post-fixed in 1% osmium tetroxide in 0.1 M sodium cacodylate buffer, rinsed again in 0.1 M sodium cacodylate buffer, dehydrated through a graded series of ethanol and propylene oxide, infiltrated through a graded series of Poly/Bed 812 (Glauert formulations) in propylene oxide, and embedded in Poly/Bed 812 (Glauert formulations). Semithin (300 nm) sections were cut using a Leica Reichert Ultracut, stained with 0.5% toluidine blue in 1% sodium borate, and then imaged under bright-field illumination on a Leica CTR5000 fluorescence microscope. Ultrathin sections (65 nm) were stained with uranyl acetate and Reynold’s lead citrate and examined on a JEOL 1400 Plus transmission electron microscope equipped with a side-mounted Nikon Digital Sight 50M camera (Nikon) at ×5,000 magnification.

Five independent fields were captured per sample. Within each field, total axons, degenerated myelin, remyelinated myelin, redundant myelin, degenerated axons, and vacuoles were counted. For each sample, the mean of the 5 independent fields was used in subsequent analyses. The investigator was blinded to the experimental groups.

AxonDeepSeg installation and analysis of captured ×5,000 transmission electron microscopy images were performed according to the instructions at https://axondeepseg.readthedocs.io/en/latest/ The generalist model was used, with morphometric parameters calculated from the image pixel size (0.00823 μm/pixel). Axons touching the image boundary or identified as having a g-ratio ≥1 were excluded from analysis. Axons with diameters <0.2 μm were manually inspected, and only structures confirmed as axons were retained. G-ratio analyses were performed using the mean values of 2 independent fields per sample, calculated either as an overall average or as averages binned by axon diameter sizes in 0.5 μm increments up to 3 μm, with all axons larger than 3 μm grouped into a final >3 μm bin. Further visualization of the relationship between the g-ratio and axon diameter was carried out using total axon counts per group in a scatter plot for descriptive visualization only.

### Correlation analyses.

*hLMNB1* mRNA expression was tested for correlation with the mRNA expression of lipid synthesis and proinflammatory genes derived from qRT-PCR data. In addition, the gene expression was tested for association with rotarod performance at 10 months of age, as a measure of disease severity.

Spearman’s rank correlation coefficient was used to assess relationships between variables. Correlations were evaluated across the combined dataset (global), including WT, TG-flox;Cre, TG-flox, with the TG-flox;Cre (3M) and TG-flox;Cre (6M) groups further combined into TG-flox;Cre. Best-fit lines were included to enhance the visualization of the association.

### Statistics.

Statistical analyses were primarily performed with GraphPad Prism 10.0 software. When appropriate, Welch’s *t* test, 1-way ANOVA with Tukey’s post hoc test, Kruskal-Wallis test with Dunn’s post hoc test, Welch’s ANOVA with Dunnett’s T3 post hoc test, mixed-effects analysis with Tukey’s post hoc test, 2-way ANOVA with Tukey’s post hoc test, and survival analysis using the log-rank (Mantel-Cox) test were used. Western blot, qRT-PCR, and biomarker data were assumed to be approximately normally distributed, and 1-way ANOVA with Tukey’s post hoc test was used.

For log-rank (Mantel-Cox) analyses, pairwise comparisons were performed, and multiple testing corrections were applied manually. When Prism reported *P* < 0.0001, a conservative *P* value of 0.0001 was used for manual correction.

Linear regression was applied to the g-ratio versus axon diameter scatter plot to estimate the slopes of the relationship for descriptive visualization only. No further statistics or group comparisons were performed with total axon counts.

Rstudio (version 2025.09.1+401) was used to generate the overall improvement heatmap and to perform association analyses using Spearman’s rank correlation coefficient, with the tidyverse, readxl, ggpubr, ggplot2, dplyr, and statix packages installed.

Statistical tests performed are described in each figure legend and in the Supporting Data Values file, which also details specific comparisons and corresponding *P* values. Unless otherwise noted in the legend, data are presented as mean ± SEM, and differences were considered statistically significant at *P* < 0.05.

### Study approval.

The Institutional Animal Care and Use Committee of the University of Pittsburgh approved all mouse procedures.

### Data availability.

Data supporting the findings of the study are available in the Supporting Data Values file and supplemental tables.

## Author contributions

NH and QSP conceived and designed the overall study with contributions from BN, TKK, and XZ. Experiments were carried out by NH, KM, KAM, BN, AM, FL, MS, and XZ. Funding was procured by QSP and TKK. NH and QSP wrote the paper with contributions from KM, MS, and XZ. All authors read and approved of the final manuscript.

## Conflict of interest

The authors have declared that no conflict of interest exists.

## Funding support

This work is the result of NIH funding, in whole or in part, and is subject to the NIH Public Access Policy. Through acceptance of this federal funding, the NIH has been given a right to make the work publicly available in PubMed Central.

- NIH grants R01NS126193, R21NS131906, R33NS104384, R33NS106087, and R01NS095884 (to QSP).
- ADLD Center grant ADLD-23-001-02 (to QSP).
- NIH/National Institute on Aging grants R01 AG083874, U24AG082930, P30 AG066468, RF1AG077474, R01 AG083156, R37 AG023651, R01 AG025516, R01 AG073267, R01 AG075336, R01 AG072641, and P01 AG025204 (to TKK).
- NIH/National Institute of Neurological Disorders and Stroke grants U01 NS131740 and U01 NS141777 (to TKK).
- NIH/National Institute of Mental Health grant R01 MH108509 (to TKK).
- Aging Mind Foundation grant DAF2255207 (to TKK).
- Department of Defense grant HT94252320064 (to TKK).
- Anbridge Charitable Fund (to TKK).
- Department of Psychiatry, University of Pittsburgh, professional endowments (to TKK).

## Supplementary Material

Supplemental data

Unedited blot and gel images

Supporting data values

## Version 1 08/11/2026

In-Press Preview

## Version 2 09/22/2026

Electronic publication
