# Supplementary material for: LMNB1 reduction is a potential therapeutic strategy in a mouse model of autosomal dominant leukodystrophy
Source: JCI Insight. 2026 Aug 11;11(18):e207889. doi: 10.1172/jci.insight.207889 (PMC13596716; doi:10.1172/jci.insight.207889)
Supplement: Supplementary file 1 [file jciinsight-11-207889-s201.pdf]

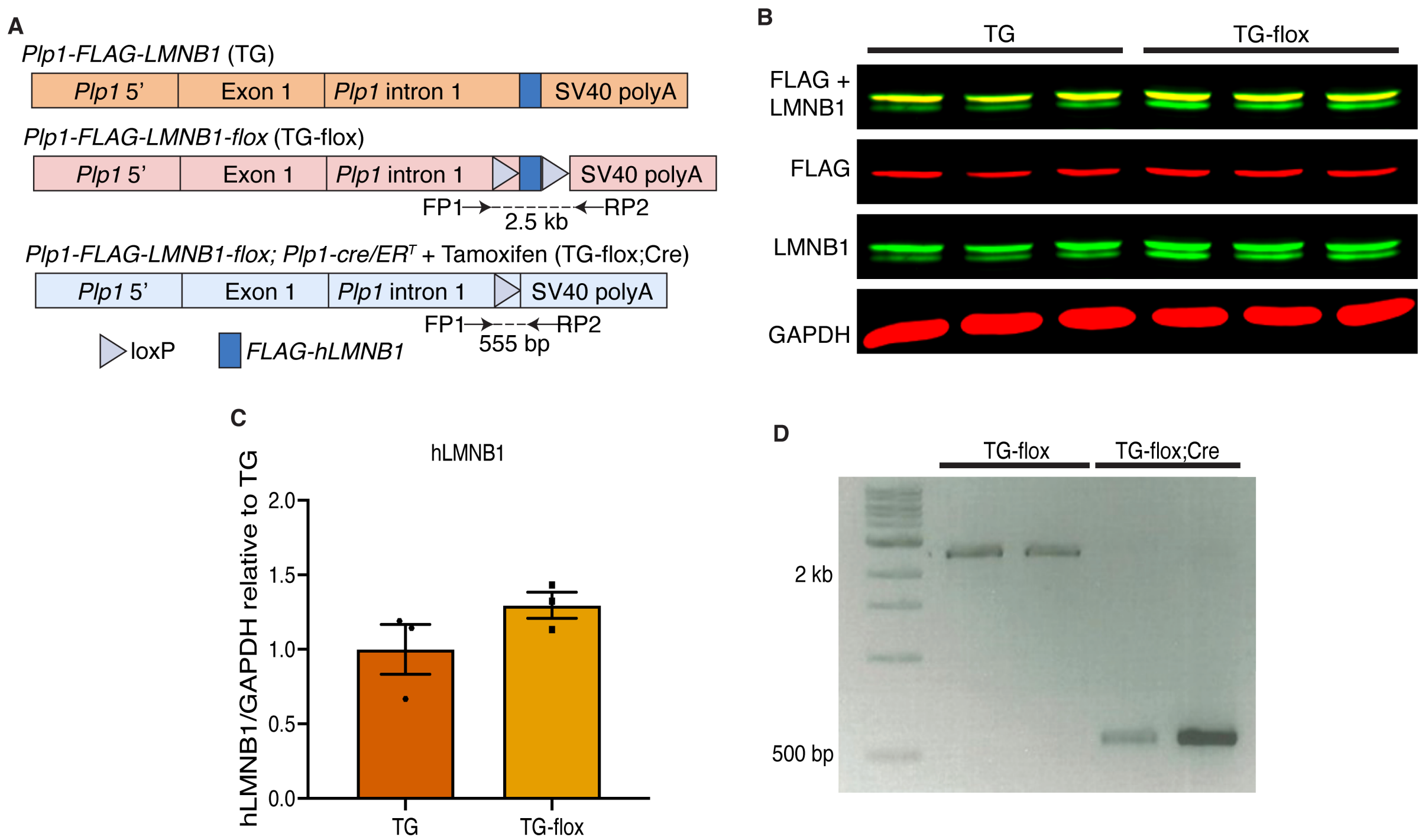

Supplemental Figure 1: Protein expression between ADLD mouse lines is similar, and excision of hLMNB1 is confirmed by gDNA PCR, related to Figure 1.

(**A**) Schematic of the *Plp-FLAG-LMNB1* (TG) and *Plp-FLAG-LMNB1*-flox cassette without (TG-flox) and with *Plp1*-cre/ER^{T} (TG-flox;Cre) + tamoxifen (TAM) injections. Arrows indicate the binding sites of the forward (FP1) and reverse (RP2) primers, as well as the expected product sizes. (**B**-**C**) Representative western blot (**B**) and quantification (**C**) of ADLD mouse line spinal cord lysates probed for endogenous LMNB1, exogenous FLAG-tagged human LMNB1 (hLMNB1), and GAPDH. hLMNB1 migrates slightly higher than endogenous mouse LMNB1. hLMNB1 levels were normalized to GAPDH and further normalized to TG. No significant differences were found between TG and TG-flox. n = 3 per group. (**D**) Spinal cord gDNA PCR using FP1 and RP2 from TAM-injected mice. (Statistical tests: B. Welch's t-test. Non-significant comparisons not shown.)

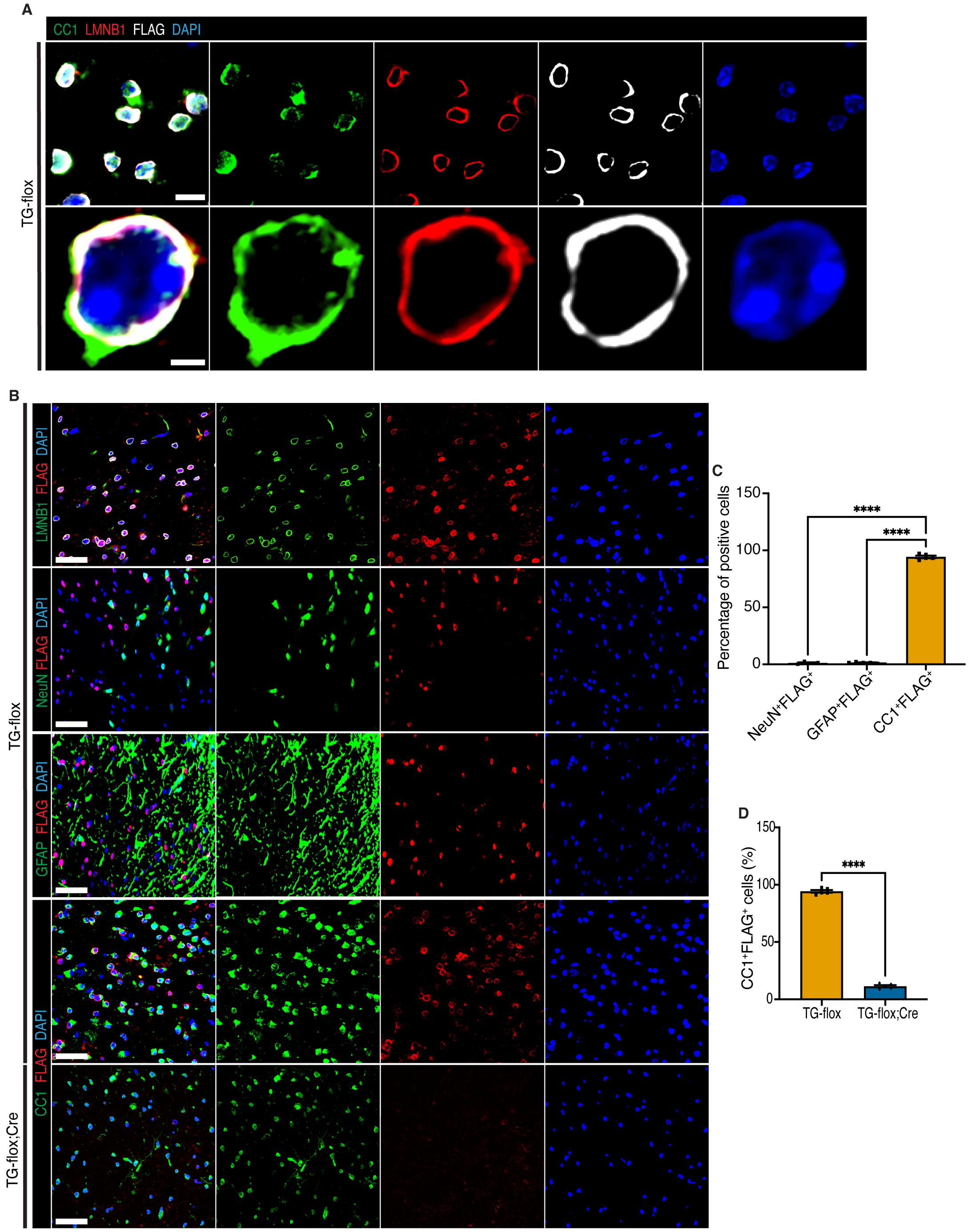

Supplemental Figure 2: Validation of oligodendrocyte-specific expression of hLMNB1 and Cre-mediated ablation.

(**A**) Representative Tg-flox spinal cord oligodendrocyte stained with CC1, LMNB1, FLAG, and DAPI. Scale bar = 10 µm (top) and 2 µm (bottom). (**B**) Representative Tg-flox or Tg-flox;Cre

spinal cord sections stained with LMNB1, NeuN, GFAP, or CC1 together with FLAG and DAPI. NeuN labels neurons, GFAP labels astrocytes, CC1 labels mature oligodendrocytes, and FLAG labels exogenous human LMNB1. Scale bar = 30 μm. (**C**) Quantification of the percentage of $NeuN^+$, $GFAP^+$, or $CC1^+$ cells that were also $FLAG^+$ in Tg-flox mice. $n \geq 4$ per group. (**D**) Quantification of the percentage of $CC1^+$ cells that were also $FLAG^+$ in Tg-flox and Tg-flox;Cre mice. $n \geq 4$ per group. All mice were TAM-injected. Each point represents one independent field per mouse. (Statistical tests: **C**. Welch's ANOVA test with Dunnett's T3 post-hoc test; **D**. Welch's t-test. Data plotted as mean ± SEM. ****$p < 0.0001$, non-significant comparisons not shown.)

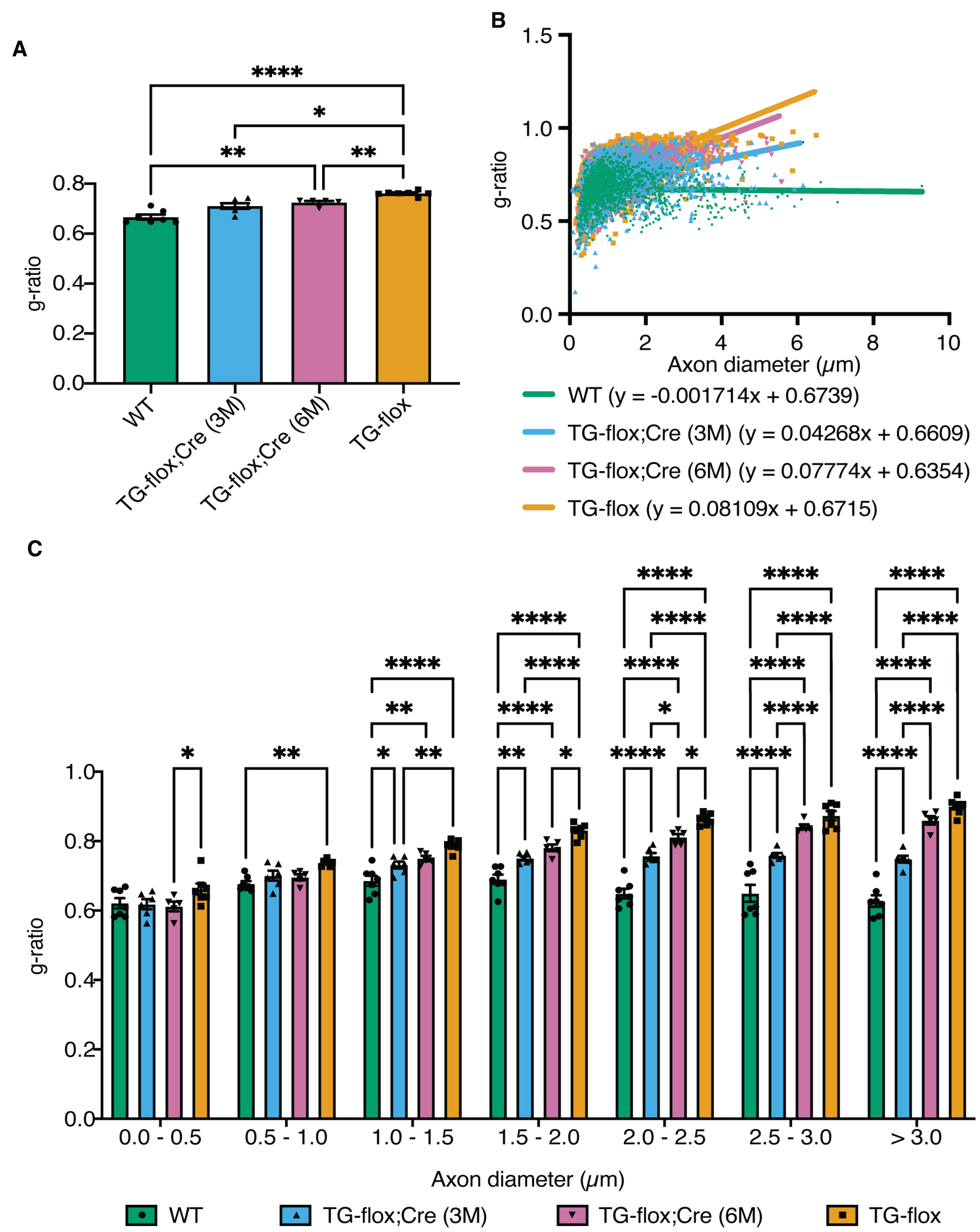

Supplemental Figure 3: AxonDeepSeg analysis shows an axon diameter-dependent increase in g-ratio within transgenic mice, related to Figure 5.

(**A**) Quantification of mean g-ratio. Each point represents the average of two independent fields per mouse. $n \geq 5$ per group. (**B**) Scatterplot of g-ratio against axon diameter for all myelinated axons with a linear regression line and its slope denoted for each group. $n \geq 1{,}540$ axons for all groups. (**C**) Quantification of mean g-ratio across axon diameter bins. Each point represents the average of two independent fields per mouse $n \geq 5$ per group. (Statistical tests: **A**. Welch's ANOVA test with Dunnett's T3 post-hoc test one-way; **B**. simple linear regression for slope

determination and visualization; **C**. Mixed effects test with Tukey's post-hoc test. Data plotted as mean ± SEM. *$p < 0.05$, **$p < 0.01$, ***$p < 0.001$, ****$p < 0.0001$, non-significant comparisons not shown.)

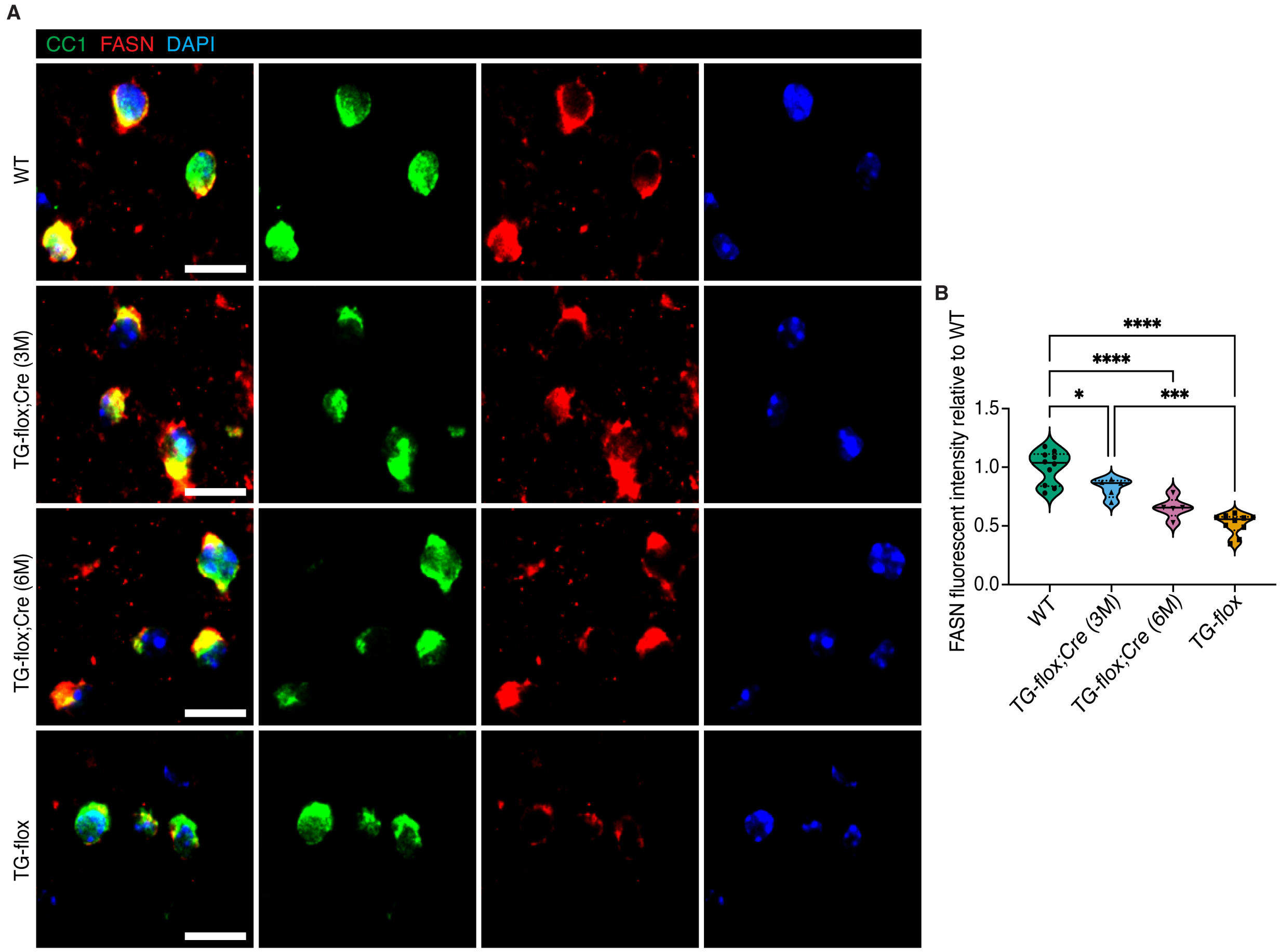

Supplemental Figure 4: FASN intensity is reduced regardless of hLMNB1 reduction time point, related to Figure 6.

(**A**) Representative spinal cord sections stained for CC1, FASN, and DAPI. CC1 labels mature oligodendrocytes, and FASN labels the fatty acid synthase protein. Scale bar = 20 µm. (**B**) Quantification of the FASN fluorescence intensity normalized to Wt. Each point represents the average of at least 16 cells per sample. $n \geq 5$ per group. (Statistical tests: **B**. one-way ANOVA test with Tukey's post-hoc test. Data plotted as mean ± SEM. $*p < 0.05$, $***p < 0.001$, $****p < 0.0001$, non-significant comparisons not shown.)

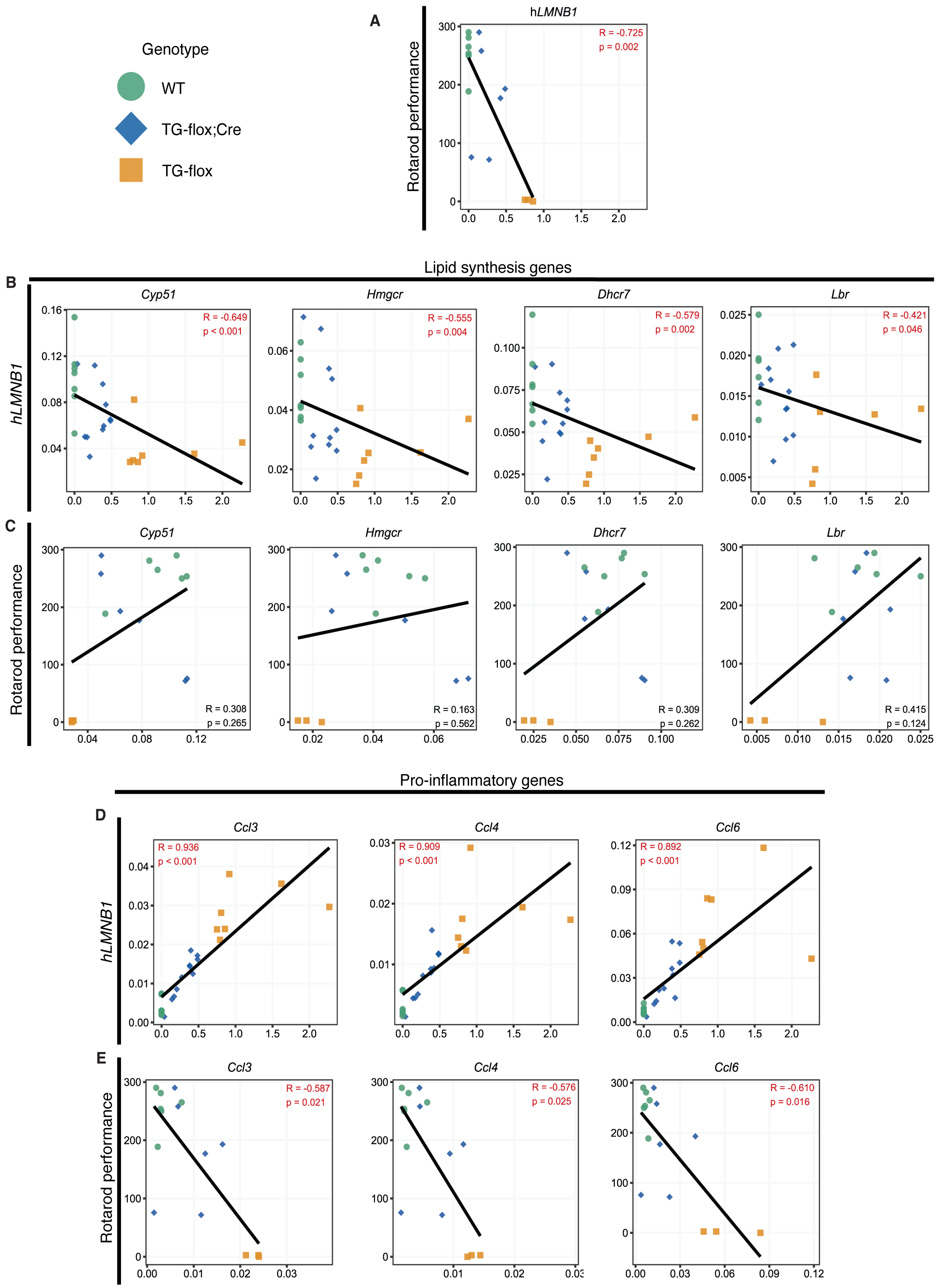

Genotype
WT
TG-flox;Cre
TG-flox
A
hLMNB1
Rotarod performance
R = -0.725
p = 0.002
Lipid synthesis genes
B
hLMNB1
Cyp51
R = -0.649
p < 0.001
Hmgcr
R = -0.555
p = 0.004
Dhcr7
R = -0.579
p = 0.002
Lbr
R = -0.421
p = 0.046
C
Rotarod performance
Cyp51
R = 0.308
p = 0.265
Hmgcr
R = 0.163
p = 0.562
Dhcr7
R = 0.309
p = 0.262
Lbr
R = 0.415
p = 0.124
Pro-inflammatory genes
D
hLMNB1
Ccl3
R = 0.936
p < 0.001
Ccl4
R = 0.909
p < 0.001
Ccl6
R = 0.892
p < 0.001
E
Rotarod performance
Ccl3
R = -0.587
p = 0.021
Ccl4
R = -0.576
p = 0.025
Ccl6
R = -0.610
p = 0.016

Supplemental Figure 5: Pro-inflammatory gene expression, but not lipid synthesis expression or neurodegeneration biomarkers, correlates with both *hLMNB1* levels and rotarod performance, related to Figure 6.

(**A**) Correlation of *hLMNB1* mRNA expression with rotarod performance at 10 months of age. *n* = 15 (WT: *n* = 6, TG-flox;Cre: *n* = 6 [3M:6M = 6:0], TG-flox: *n* = 3). (**B**-**C**) Correlation of lipid synthesis genes (*Cyp51*, *Hmgcr*, Dhcr7, and *Lbr*) with *hLMNB1* expression (**B**) or rotarod performance at 10 months of age (**C**). *n* = 25 (WT: *n* = 7, TG-flox;Cre: *n* = 11 [3M:6M = 6:5], TG-flox: *n* = 7) (**B**), *n* = 15 (WT: *n* = 6, TG-flox;Cre: *n* = 6 [3M:6M = 6:0], TG-flox: *n* = 3) (**C**). (**D**-**E**) Correlation of pro-inflammatory genes (*Ccl3*, *Ccl4*, *Ccl6*) with *hLMNB1* expression (**D**) or rotarod performance at 10 months of age (**E**). *n* = 25 (WT: *n* = 7, TG-flox;Cre: *n* = 11 [3M:6M = 6:5], TG-flox: *n* = 7) (**D**), *n* = 15 (WT: *n* = 6, TG-flox;Cre: *n* = 6 [3M:6M = 6:0], TG-flox: *n* = 3) (**E**). Solid lines indicate best-fit lines for visualization. Spearman's rank correlation coefficients (*R*), and p-values are shown within each panel. Red comparisons indicate statistical significance.

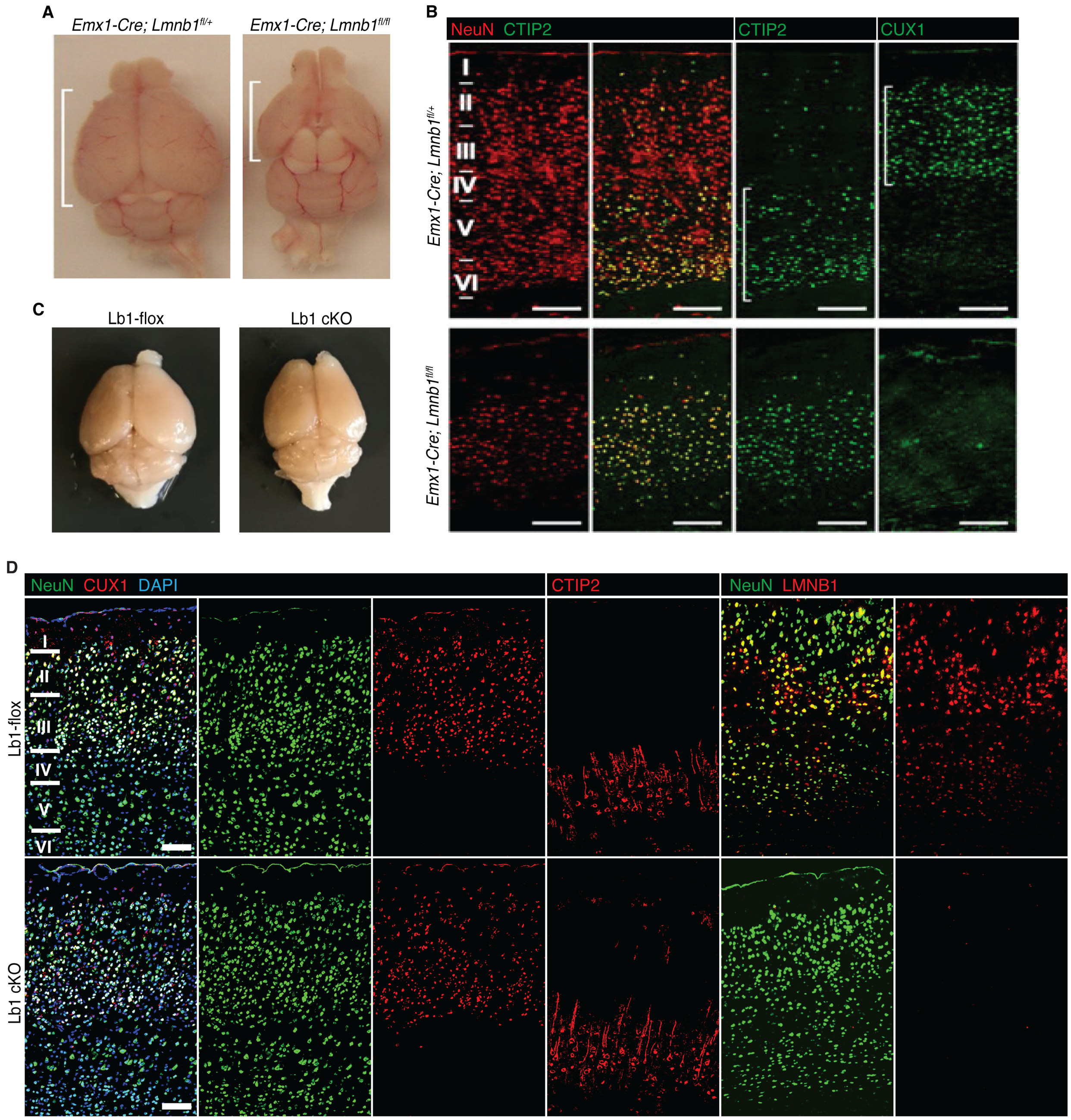

Supplemental Figure 6: Embryonic forebrain-specific knockout of *Lmnb1* results in decreased forebrain size and abnormal cortical neuron layering, related to Figure 8. Adapted from PMID: 21976703.

Panels **A** and **B** are adapted from previously published work using an Emx1-Cre mediated embryonic, forebrain-specific *Lmnb1* knockout *(*PMID: 21976703). Panels **C**-**E** show data

generated in this study using an inducible, tissue-wide UBC-Cre-mediated *Lmnb1* knockout in adulthood. Panels **C**–**E** are reproduced from our Fig. 8J–K to facilitate direct comparison with the embryonic, forebrain-specific *Lmnb1* knockout.

(**A**) Representative images of a 1-month-old *Emx1-Cre* mouse forebrain, with the forebrain region indicated by brackets. (**B**) Representative cortical sections from 1-month-old *Emx1-Cre* mice stained with NeuN, CUX1, CTIP2, and DAPI. NeuN labels neurons, CUX1 labels layers II to upper IV, and CTIP2 labels lower IV to VI. Cortical layers are labeled with Roman numerals. Scale bar = 200 µm. (**C**) Representative forebrain images from 12-month-old UBC-Cre mice. (**D**) Representative cortical sections stained with a combination of NeuN, CUX1, DAPI, CTIP2, or LMNB1 antibodies. Cortical neuron layers are labeled by roman numerals in Lb1-flox images. Scale bar = 50 µm.

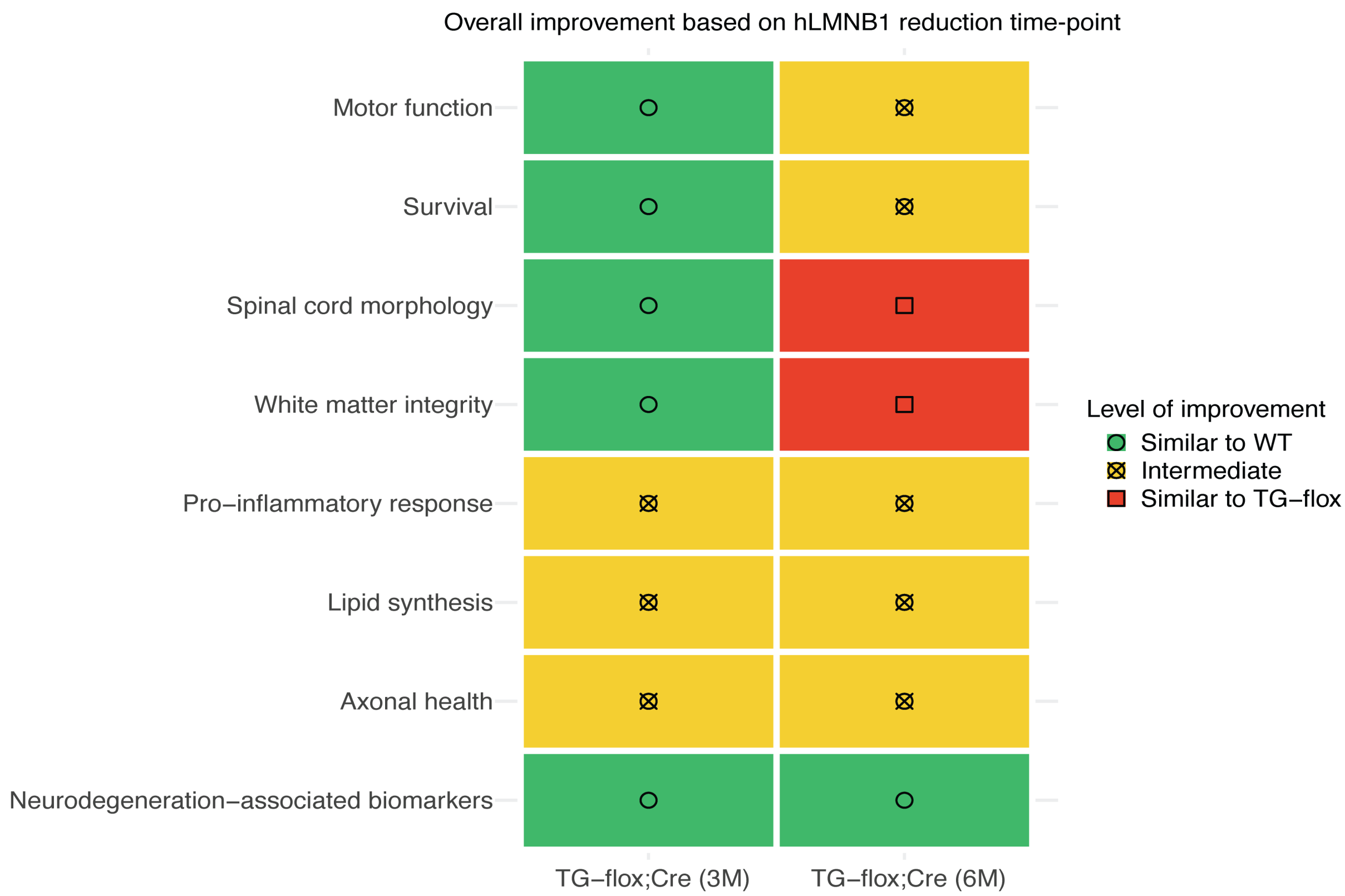

Supplemental Figure 7: Early reduction of hLMNB1 leads to greater overall improvement compared to late reduction

Heatmap showing the level of improvement in TG-flox;Cre (3M) and TG-flox;Cre (6M) across assessed categories. "Similar to WT" or "Similar to TG-flox" indicates that the cohort was indistinguishable from them, while 'intermediate' indicates partial improvement over TG-flox or mixed results across measures.

Supplemental Table 1: Functional observation battery (FOB) scoring criteria.

Assessment Criteria		0	1	2
Observation	In cage motor activity/frequency	Moves frequently	Limited mobility	Immobile, even upon touch
	Gait	Normal	Mild ataxia	Severe ataxia or paralysis
	Condition of fur	Smooth, shiny coat	Ungroomed or patchy fur	Matted fur, piloerection
	Posture/body tone	Normal, relaxed posture	Mild to moderate hunching	Severe hunching
	Seizures	None observed	Seizures upon handling	Continuous seizures
	Tremors	None observed	Mild tremors	Continuous tremors
	Eyelids/ptosis	Normal	Partial ptosis	Complete ptosis
Handling	Tail flip test	Cannot flip mouse	Flips, but reverts upright	Flips, but cannot regain position
	Tail pinch	Immediate response	Delayed response	No response
	Grip test	Strong grip on mesh	Weak grip	No response

Supplemental Table 2: Sequences of oligonucleotides used in the study.

Genotyping primers

	Primer sequences	Expected sizes
Plp-FLAG-LMNB1-flox	Forward: TAACATGGGCTGCATAACTTCG<br>Reverse: TCTTAGCATAGTTGAGGAGCAGC	Transgenic: 550 bp<br>Wild-type: 324 bp
Plp1-cre/ER^{T}	Forward: AGGTGGACCTGATCATGGAG<br>Reverse: ATACCGGAGATCATGCAAGC	Transgenic: 440 bp<br>Wild-type: 324 bp
UBC-Cre-ER^{T2}	Forward: AGGCGTTTTCTGAGCATACCTG<br>Reverse: AACATTCTCCCACCGTCAGTAC	Transgenic: 478 bp<br>Wild-type: 324 bp
Lmnb1-flox	Flox Forward: AACAAACTTGGCCTCACCAG<br>Wild-type Forward: CCACTTAGCTCGGGGAGTTT<br>Common Reverse: CTGTGGGACAAAGACCCAGT	Transgenic: 450 bp<br>Wild-type: 300 bp
Gapdh (internal control)	Forward: GGCCTGTTTAAGGGAAAGAAGT<br>Reverse: CCCCTTCATTGACCTCAACTAC	-

** Gapdh was used with Plp-FLAG-LMNB1-flox, PLP/creERT, and UBC-Cre-ERT2 primers as an internal control for genotyping*

Knockout gDNA primers

	Primer sequences	Expected sizes
hLMNB1 knockout	Forward Primer 1 (FP1): CCTTTTACTTTTCCCATGGTCTCC<br>Reverse Primer 2 (RP2): TTCCGAAGCCCAACCTTTCATA	Intact: 2542 bp<br>Knockout: 555 bp

mRNA expression qRT-PCR primers

	Primer sequences
Mouse *β-Actin*	CCACTGCCGCATCCTCTTCC<br>CTCGTTGCCAATAGTGATGACCTG
Mouse *Lmnb1*	GCTGCTCAATTATGCCAAGAAG<br>GCCGCATCCTTAGAGTTTAGT
Human *LMNB1*	GATTGCCCAGTTGGAAGCCT<br>TGGTCTCGTTAATCTCCTCTTCATACA
Mouse *Cyp51*	GGCCTTAACATAGCCCACTT<br>CACATTTCTTTCTCCGCTTTCTC
Mouse *Hmgcr*	CCAGAAGCTTTCGTCAGTAGAG<br>CTCTGCTTGTAGTCTCTGCTTC
Mouse *Dhcr7*	CCTGACTTCTGCCACAGATT<br>CAGCCCATTCACCTCATACTT
Mouse *Lbr*	CCCGAACACATGGAGAAGAA<br>CGAAGGCTGTACTGTGTAACT
Mouse *Ccl3*	GAAGATTCCACGCCAATTCATC<br>GATCTGCCGGTTTCTCTTAGTC
Mouse *Ccl4*	CCACTTCCTGCTGTTTCTCTTA<br>CAAAGACTGCTGGTCTCATAGT
Mouse *Ccl6*	CGTCGCTATAACCCTCCAATAA<br>TGGGATCTGTGTGGCATAAG

Supplemental Table 3: List of antibodies used in the study.

Western blot antibodies

	Antibody	Species	Dilution	Manufacturer	Catalog #
Primary	LMNB1	Rabbit	1:1,500	Abcam	ab16048
	FLAG	Mouse	1:1,500	Sigma-Aldrich	F1804
	β-ACTIN	Mouse	1:3,000	ThermoFisher	MA5-15739
	GAPDH	Mouse	1:5,000	ThermoFisher	MA5-15738
Secondary	Anti-Mouse IRDye 680LT	Goat	1:10,000	LI-COR Biosciences	926-68020
	Anti-Rabbit IRDye 800CW	Donkey	1:10,000	LI-COR Biosciences	926-32213

Immunofluorescence antibodies

	Antibody	Species	Dilution	Manufacturer	Catalog #
Primary	LMNB1	Rabbit	1:500	Abcam	ab16048
	FLAG	Mouse	1:100	Sigma-Aldrich	F1804
	FLAG	Rabbit	1:200	Sigma-Aldrich	F7425
	NeuN	Mouse	1:100	Millipore	MAB377
	GFAP	Mouse	1:250	Millipore	MAB360
	MBP	Chicken	1:500	EnCor	CPCA-MBP
	PLP	Rat	1:100	Millipore	MABN2620
	CC-1 (Anti-APC)	Mouse	1:100	Abcam	AB16794-1002
	APP	Rabbit	1:100	Invitrogen	512700
	IBA1	Rabbit	1:250	Wako Chem	019-19741
	FASN	Rabbit	1:200	Abcam	ab22759
	CUX1	Rabbit	1:500	ProteinTech	11733-1-AP
	CTIP2 (BCL11B)	Rabbit	1:200	ProteinTech	55414-1-AP
Secondary	Anti-Chicken FITC	Donkey	1:350	Jackson IR	703-095-155
	Anti-Rat Cy3	Donkey	1:350	Jackson IR	712-165-150
	Anti-Mouse FITC	Donkey	1:350	Jackson IR	715-096-150
	Anti-Mouse Cy3	Donkey	1:350	Jackson IR	715-165-150
	Anti-Rabbit Cy3	Donkey	1:350	Jackson IR	711-165-152
	Anti-Rabbit FITC	Donkey	1:350	Jackson IR	711-095-152
