# Supplementary material for: LMNB1 reduction is a potential therapeutic strategy in a mouse model of autosomal dominant leukodystrophy
Source: JCI Insight. 2026 Aug 11;11(18):e207889. doi: 10.1172/jci.insight.207889 (PMC13596716; doi:10.1172/jci.insight.207889)
Supplement: Supplementary file 2 [file jciinsight-11-207889-s202.pdf]

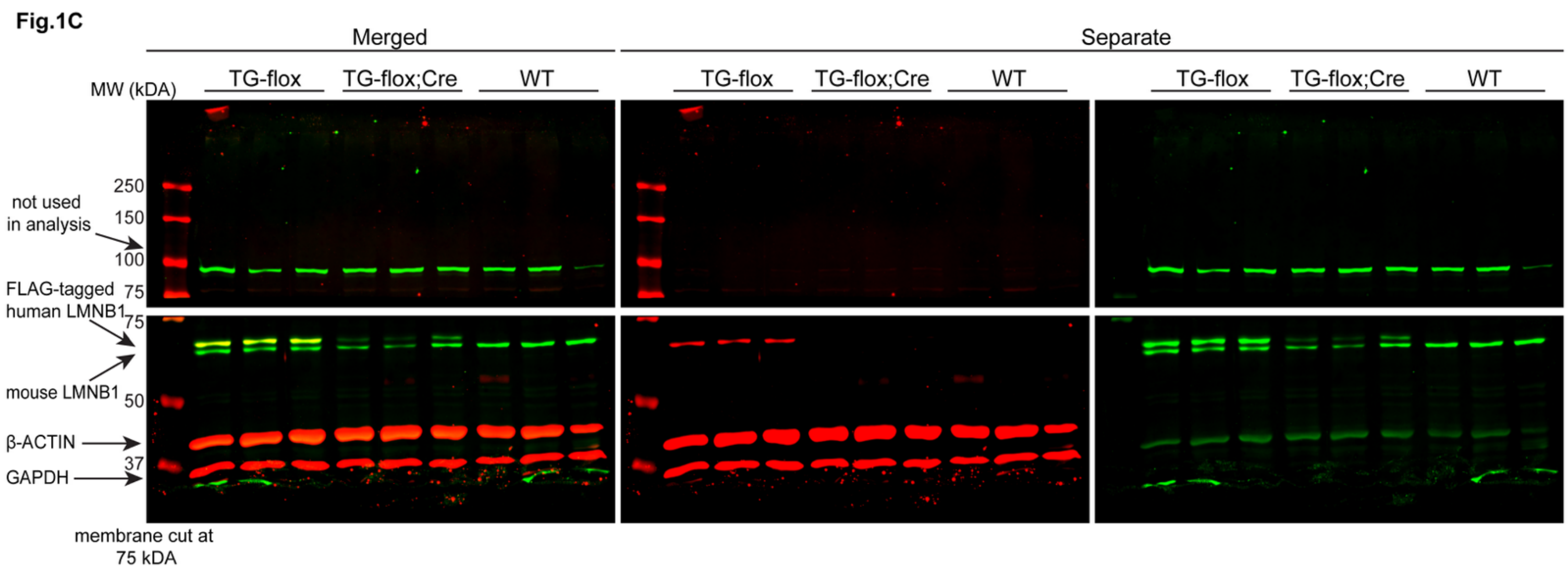

Uncropped western blots corresponding to Fig. 1C. Bands not labeled represent non-specific signals.

Fig.8A - Forebrain

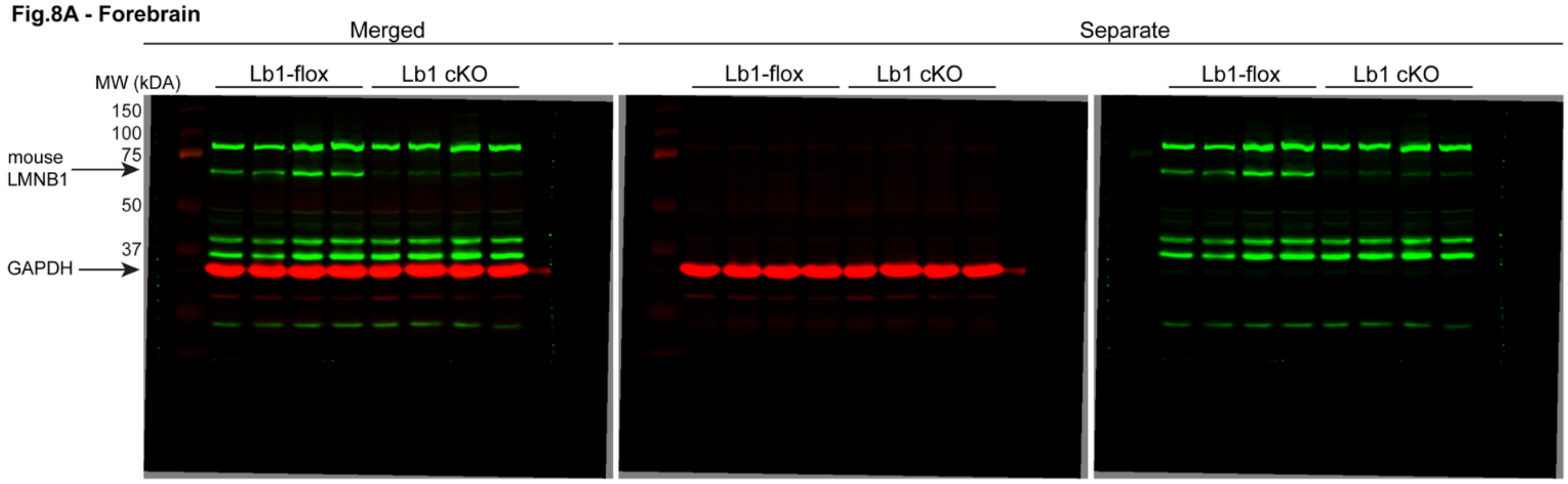

Fig.8A - Spinal Cord

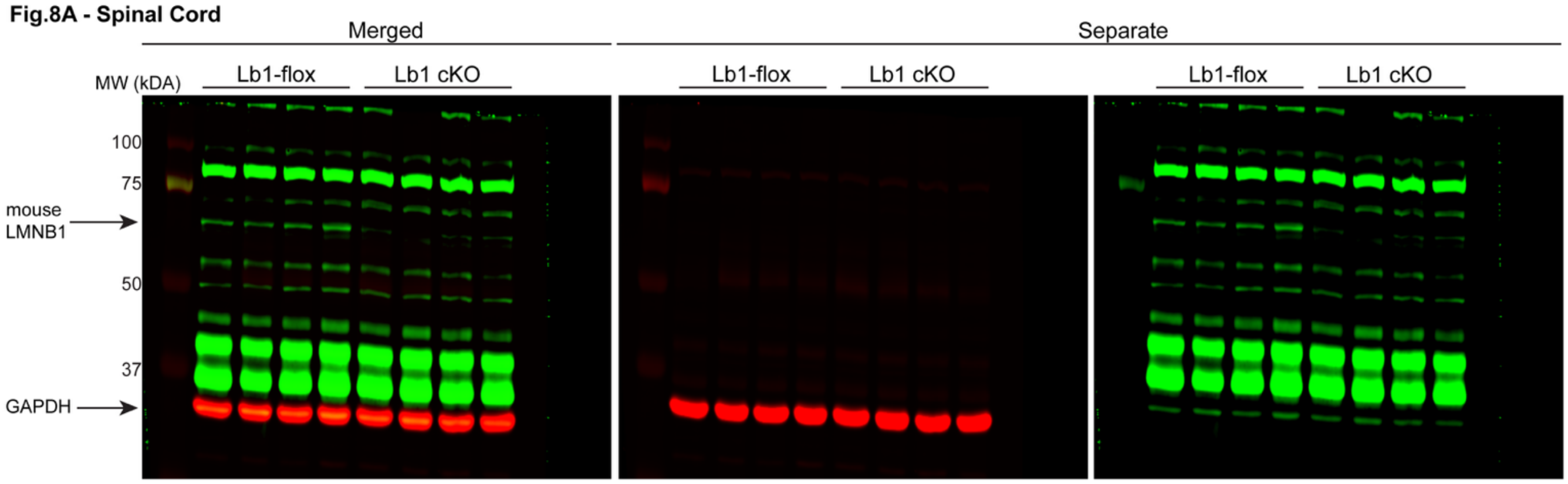

Uncropped western blots corresponding to Fig. 8A. Bands not labeled represent non-specific signals.

Supplemental Fig.1B

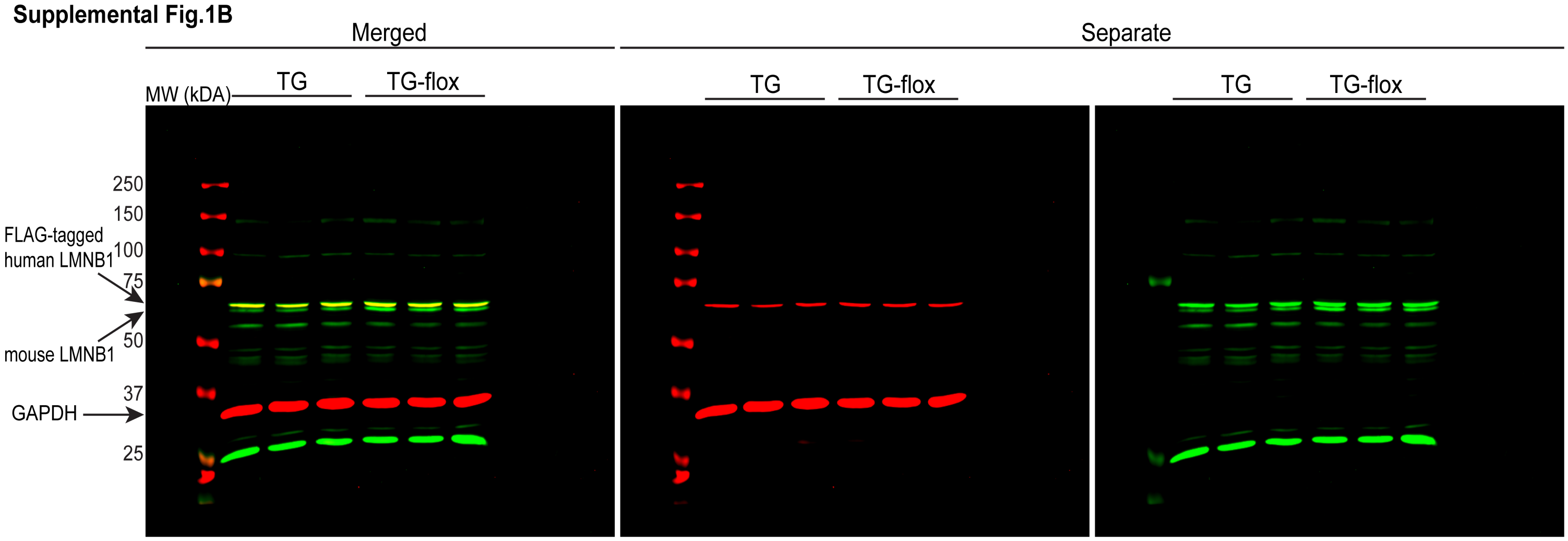

Uncropped western blots corresponding to Supplemental Fig. 1B. Bands not labeled represent non-specific signals.
